# Phase III randomised trial comparing 6 vs. 12-month of capecitabine as adjuvant chemotherapy for patients with stage III colon cancer: final results of the JFMC37-0801 study

**DOI:** 10.1038/s41416-019-0410-0

**Published:** 2019-03-05

**Authors:** Naohiro Tomita, Katsuyuki Kunieda, Atsuyuki Maeda, Chikuma Hamada, Takeharu Yamanaka, Toshihiko Sato, Kazuhiro Yoshida, Narikazu Boku, Riichiro Nezu, Shigeki Yamaguchi, Hideyuki Mishima, Sotaro Sadahiro, Kei Muro, Megumi Ishiguro, Junichi Sakamoto, Shigetoyo Saji, Yoshihiko Maehara

**Affiliations:** 1 Department of Surgery, Division of Lower Gastrointestinal Surgery, Hyogo College of Medicine, Hyogo, Japan; 2 Department of Surgery, Gifu Prefectural General Medical Center, Gifu, Japan; 3 Department of Surgery, Ogaki Municipal Hospital, Gifu, Japan; 4 Graduate School of Engineering, Tokyo University of Science, Tokyo, Japan; 5 Department of Biostatistics, Yokohama City University School of Medicine, Kanagawa, Japan; 6 Yamagata Prefectural Central Hospital, Yamagata, Japan; 7 Department of Surgical Oncology, Gifu University, Graduate School of Medicine, Gifu, Japan; 8 National Cancer Center Hospital, Tokyo, Japan; 9 Nishinomiya Municipal Central Hospital, Hyogo, Japan; 10 Department of Gastroenterological Surgery, Saitama Medical University International Medical Center, Saitama, Japan; 11 Cancer Center, Aichi Medical University, Aichi, Japan; 12 Department of Surgery, Tokai University School of Medicine, Kanagawa, Japan; 13 Aichi Cancer Center, Aichi, Japan; 14 Department of Translational Oncology, Tokyo Medical and Dental University Graduate School, Tokyo, Japan; 15 Japanese Foundation for Multidisciplinary Treatment of Cancer, Tokyo, Japan; 16 Japanese Foundation for Multidisciplinary Treatment of Cancer, Tokyo, Japan; 17 Japanese Foundation for Multidisciplinary Treatment of Cancer, Tokyo, Japan

**Keywords:** Colon cancer, Randomized controlled trials

## Abstract

**Background:**

Up to 6-months oxaliplatin-containing regimen is now widely accepted as a standard adjuvant chemotherapy for stage III colorectal cancer (CRC). However, oral fluoropyrimidine monotherapy is used for some part of patients, especially in Asian countries including Japan, and its optimal duration is yet to be fully investigated.

**Methods:**

A total of 1306 patients with curatively-resected stage III CRC were randomly assigned to receive capecitabine (2500 mg/m^2^/day) for 14 out of 21 days for 6 (*n* = 654) or 12 (*n* = 650) months. The primary endpoint was disease-free survival (DFS), and the secondary endpoints were relapse-free survival (RFS), overall survival (OS), and adverse events.

**Results:**

The 3- and 5-year DFS were 70.0% and 65.3% in the 6M group and 75.3% and 68.7% in the 12M group, respectively (*p* = 0.0549, HR = 0.858, 90% CI: 0.732–1.004). The 5-year RFS was 69.3% and 74.1% in the 6M and 12M groups, respectively (*p* = 0.0143, HR = 0.796, 90% CI: 0.670–0.945). The 5-year OS was 83.2% and 87.6%, respectively (*p* = 0.0124, HR = 0.727, 90% CI: 0.575–0.919). The incidence of overall grade 3–4 adverse events was almost comparable in both groups.

**Conclusions:**

Although 12-month adjuvant capecitabine did not demonstrate superior DFS to that of 6-month, the observed better RFS and OS in the 12-month treatment period could be of value in selected cases.

## Background

Colorectal cancer (CRC) is among the most common malignancies worldwide. Annually, >1,350,000 new cases are diagnosed, and approximately 700,000 people die from this disease.^1^ In Japan, the incidence of CRC cases has increased recently, with approximately 149,500 new CRC cases reported in 2017.^2^

Surgery is the main treatment for CRC, while postoperative adjuvant chemotherapy is used to reduce recurrence and improve prognosis. Three previous studies—the NCCTG 894651,^3^ INT-0089^4^ and another^5^ studies—evaluated drug selection and administration for postoperative adjuvant chemotherapy for CRC. All these studies showed no significant differences in disease-free survival (DFS) between 6 month and longer duration of 5-FU-based regimens. In the X-ACT^6^ and NSABPC-06^7^ studies, capecitabine and UFT-LV, respectively, were compared with 5-FU/LV for 6 months. Based on these studies, 6 months was determined to be the standard treatment period for postoperative adjuvant chemotherapy for CRC.

However, given the lack of sufficient detection sensitivity during the treatment periods and the differences in the treatment schedules among these studies, the National Cancer Institute Physician Data Query stated that the current evidence supporting this treatment period is not definitive.^8^ Moreover, a meta-analysis indicated that 1-year administration of oral 5-FU in stage III CRC achieved significantly superior DFS and overall survival (OS) to surgery alone,^9^ and the period of administration for oral 5-FU was set as ≥1 year in numerous Japanese clinical trials. Thus, whether 6 months is the optimal treatment period for oral 5-FU drugs as the adjuvant postoperative chemotherapy for CRC remains inconclusive.

By analysing the year-to-year hazard rate of recurrence after curative surgery, Hamada et al.^10^ recently suggested that prolonged administration of oral 5-FU might improve the prognosis of patients with CRC. The recurrence risk in the three study groups peaked between 1 and 2 years postoperatively. However, no peak recurrence risk was observed in the 1-year oral 5-FU drug therapy (drugs used: UFT and HCFU) group.

Although 6-month adjuvant chemotherapy is the current standard treatment modality for patients with stage III CRC, whether prolonged chemotherapy, particularly using oral fluoropyrimidine, can improve patient survival remains unclear.

Aside from fluoropyrimidines, the new agent oxaliplatin has become one of the primary adjuvant chemotherapeutic for CRC. Based on the results of three randomised controlled trials—MOSAIC,^11^ NSABP C-07,^12^ and XELOXA^13^—infusional fluorouracil and folinic acid with oxaliplatin (FOLFOX) and capecitabine with oxaliplatin (CapeOX) have become the standard postoperative adjuvant chemotherapy for CRC.^14^ Six-month oxaliplatin-based treatment might be an ideal control arm to investigate the potential role of prolonged administration of 5-FU in randomised controlled studies. However, in Japan, oxaliplatin could not be used when the current trial was designed because it only became available in August 2009. Therefore, we adopted 6-month capecitabine as the standard treatment for investigating the clinical utility of prolonged oral 5-FU administration in the adjuvant setting.

Herein, we report the final results of our multi-institutional randomised controlled trial comparing 6 vs. 12 months of capecitabine as adjuvant chemotherapy for patients with stage III CRC (JFMC37-0801).

The safety and feasibility of the 12-month capecitabine regimen in this study was previously reported.^15^

## Patients and methods

### Study design

JFMC37-0801 was a multi-institutional, open-label, randomised, phase III study (see Supplementary Figure 1). It was conducted in accordance with the Declaration of Helsinki and the Ethical Guidelines for Clinical Research in Japan and was approved by the Institutional Review Boards of each participating institute. Written informed consent was obtained from all patients before their enrolment, and then the eligible patients were centrally registered. This study primarily aimed to demonstrate the superiority of adjuvant 12 months of capecitabine (16 courses) to 6 months (eight courses) in terms of DFS for stage III (Dukes’ C) CRC after curative resection. Secondary endpoints were relapse-free survival (RFS), OS, and safety.

A complete study protocol of JFMC-37 trial is shown in Supplementary file.

### Enrolment and assignment

The main eligibility criteria were as follows: (1) histologically confirmed stage III colon adenocarcinoma; (2) curatively resected with extended lymph node dissection (D2 or D3 in the Japanese Classification of Colorectal Carcinoma, 7th edition);^15^ (3) aged 20–79 years; (4) Eastern Cooperative Oncology Group performance status (ECOG-PS) of 0–1; (5) no prior chemotherapy or radiotherapy for CRC; (6) no other active malignancies; (7) adequate oral intake; (8) preserved major organ functions and, (9) no uncontrollable severe infection.

### Randomisation and masking

After confirming eligibility, enrolled patients were randomly assigned to receive either 6 (eight cycles) or 12 (16 cycles) months of capecitabine using a minimisation method, with stratification according to lymph node metastasis (N1 or N2-3 in the Japanese Classification of Colorectal Carcinoma, 7th edition)^16^ and institution. The assigned treatment arm was not blinded from both investigators and patients.

### Protocol treatment

Oral capecitabine was administered at a dose of 1250 mg/m^2^ twice daily after meals for 14 consecutive days, followed by a 7-day rest. This 3-week treatment comprised one course. The control group (6M) and study group (12M) received 8 and 16 courses, respectively. The assigned treatment was started within 8 weeks after surgery. During treatment, clinical findings and laboratory values were evaluated at least every 3 weeks. Evaluation at the beginning of each cycle was mandatory. Patients received treatment if they fulfilled the following criteria: leucocytes ≥3000/mm^3^, neutrophils ≥1500/mm^3^, platelets ≥75,000/mm^3^, aspartate aminotransferase and alanine aminotransferase ≤ 2.5 × upper limit of normal (ULN), total bilirubin ≤ 1.5 ULN, creatinine < 1.5 × ULN, and no > grade 1 non-haematologic toxicities (i.e., anorexia, nausea, vomiting, and diarrhoea). If the criteria for starting/continuing treatment course were not fulfilled, treatment was postponed or temporarily suspended until adverse events (AEs) improved sufficiently to meet the criteria.

Depending on the severity of AEs, the dose of capecitabine was reduced according to the protocol. When a grade 2 AE developed for the first time, treatment with capecitabine was suspended until the AE improved to grade ≤ 1, and then resumed at the same dose. If a grade 2 AE occurred twice or more and if a grade 3 AE occurred, the dose of capecitabine was reduced by 25%. The minimum dose of 50% of the initial dose was allowed in the protocol.

The treatment was discontinued in the following: (1) consent was withdrawn; (2) treatment was requested to be discontinued; (3) recurrence of the original disease; (4) newly detected cancerous lesions (duplicated cancer or multifocal CRC [excluding T1a cancer]); (5) as judged by the attending physician due to deterioration of AE, comorbidities, onset of complications, or other reasons; (6) AE requiring dose delay (>3 weeks) beyond allowance pre-specified in the protocol; (7) AE requiring dose reduction > 50%; (8) Grade 4 AE; (9) death; (10) transfer to other hospital; (11) ineligibility after enrolment. If capecitabine treatment is restarted after protocol termination, documentation must be submitted.

### Primary/secondary endpoint analyses and statistical basis

The primary endpoint was DFS, which is defined as survival from the randomisation without the following events; (1) occurrence of secondary cancer (including CRCs and any other malignancies), (2) recurrence, and (3) death due to any cause. The secondary endpoints included RFS, OS, and safety. RFS was defined as the period without (2) and (3), and OS was without (3). Patients with no events at the final data cut-off were censored.

The DFS, RFS, and OS were estimated by group using the Kaplan–Meier method. Greenwood’s formula was used for interval estimation. The stratified log-rank test was used to evaluate the hypothesis. The stratified Cox proportional hazard model will be used to calculate hazard ratio. The hazard ratio of the treatment effect among the groups and its 90% confidence interval will also be obtained.

A Wald-type estimator was used to estimate confidence intervals. An adjusted hazard ratio accounting for other background factors would be derived as necessary. A similar secondary analysis will be performed for all eligible or treated subjects. As for secondary endpoint analyses, RFS and OS were evaluated using the same analysis as DFS. Chi-square test was used to compare the incidence of AEs between the treatment groups.

A one-tailed *p* < 0.0477 was considered significant in the primary endpoint analysis. Because we conducted the interim analysis at information time 0.53125 (255/480 events), 0.0022 of alpha error was spent. Therefore, if the one-sided significance level would be <0.0478, the 12M duration would be the standard therapy. In other tests, *p* < 0.05 was considered significant. All statistical analyses were performed using SAS software version 9.3 (SAS Institute, Cary, NC, USA). Descriptive statistics were calculated.

### Subjects and statistical basis

There should be no difference in DFS for first 6-month period because the 6M and 12M groups received the same treatment after randomisation, with differences expected to appear 6 months or later. When calculating the number of subjects assuming a normal exponential distribution without considering the above, the number of required subjects is underpowered. Therefore, in this study, the number of subjects was set assuming a segmented exponential model.

In the 6 months following randomisation, the hazard ratio for DFS groups would be 1.0 and assuming that differences in DFS would begin to appear after 6 months or later. Presupposing that the 5-year DFS rate in the 6M and 12M groups would be 60% and 67%, respectively, 1142 subjects (480 events) were required, with a one-sided significance level of 5% and a detection power of 80%. Finally, the number of target subjects are determined to be 1200 (600 patients per group), assuming that ~5% will drop out.

## Results

### Patient characteristics

**Fig. 1:**
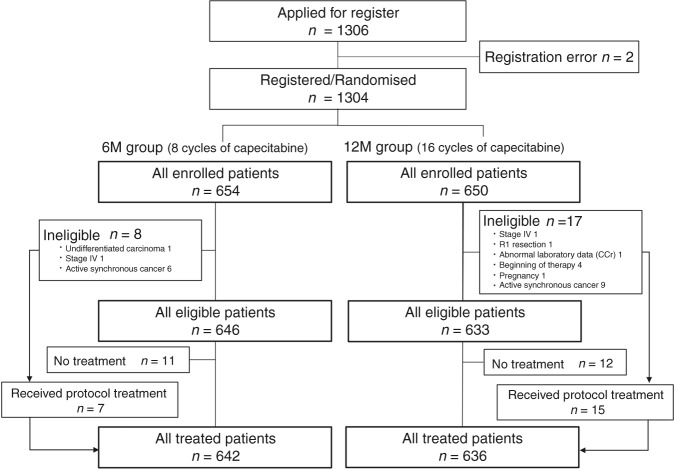
CONSORT diagram

A total of 1306 patients were enrolled from 333 institutions in Japan between September 2008 and December 2009. Excluding two patients due to registration error, 1304 patients were randomly assigned to the two groups: 654 patients in the 6M group (control arm) and 650 patients in the 12M group (study arm). Eight and 17 patients were ineligible and 11 and 12 did not receive the protocol treatment in the 6M and 12M groups, respectively. The reasons for ineligibility are provided in Fig. 1.

Based on a scheduled intention-to-treat analysis, all enrolled patients including those who were ineligible were included in the final efficacy analysis set. Patient demographics were well balanced in the two groups (Table 1). A total of 1278 patients (642: 6M group; 636: 12M group) who finally received capecitabine were included in the safety analysis set. All data for the safety analyses were finalised in March 2016.

**Table 1:** Baseline patient characteristics (*N* = 1304 intension-to-treat population)

Total number		6M group	12M group
		*n* = 654	*n* = 650
Nodal status	N1	504 (77.1%)	498 (76.6%)
	N2/N3	150 (22.9%)	152 (23.4%)
Gender	Male	352 (53.8%)	343 (52.8%)
	Female	302 (46.2%)	307 (47.2%)
Age	<70	451 (69.0%)	442 (68.0%)
	≧70	203 (31.0%)	208 (32.0%)
Tumour location	Right-sided colon (C/A/T)	262 (40.1%)	263 (40.5%)
	Left-sided colon (D/S)	258 (39.4%)	252 (38.8%)
	Rectosigmoid colon	134 (20.5%)	135 (20.8%)
Surgical approach	Laparoscopic	276 (42.2%)	255 (39.2%)
	Open(conventional)	378 (57.8%)	395 (60.8%)
Histological type	Pap/ Well	189 (28.9%)	178 (27.4%)
	Tub/Mod	419 (64.1%)	433 (66.6%)
	Poor/Solid/Mon-solid/Muc/Sig/Other	46 (7.0%)	39 (6.0%)
T(TMN 7^th^)	T1/T2	101 (15.4%)	100 (15.4%)
	T3	366 (56.0%)	363 (55.8%)
	T4	187 (28.6%)	187 (28.8%)
N (TNM 7^th^)	N1	512 (78.3%)	506 (77.8%)
	N2	142 (21.7%)	144 (22.2%)
Stage (TNM 7^th^)	IIIA	94 (14.4%)	96 (14.8%)
	IIIB	461 (70.5%)	458 (70.5%)
	IIIC/IVA/IVB	99 (15.1%)	96 (14.8%)

### Disease-free survival

**Fig. 2:**
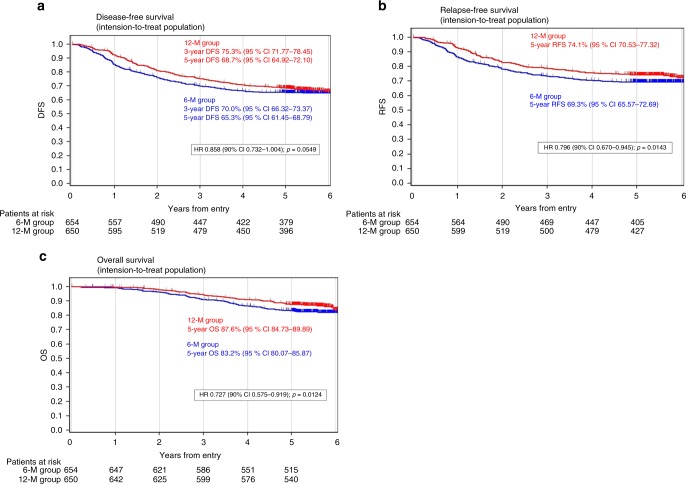
**a** Disease-free survival rate in all enrolled patients. **b** Relapse-free survival rate in all enrolled patients. **c** Overall survival rate in all enrolled patients

DFS was analysed based on 60.6 months of median follow-up with 434 events (226 and 208 events in the 6M and 12M groups, respectively). The 5-year DFS rate was 65.3% (95% CI: 61.45–68.79) and 68.7% (95% CI: 64.92–72.10) in the 6M and 12M groups, respectively. The HR was 0.858 (90% CI: 0.732–1.004; *p* = 0.0549). No statistical difference in DFS was noted between the 12M and 6M group with the significance level of 0.0478 (Fig. 2a, Table 2). HR in all eligible and treated patients was 0.833 (90% CI: 0.710–0.979; *p* = 0.0308) and 0.848 (90% CI: 0.722–0.995; *p* = 0.0452), respectively.

**Table 2:** Efficacy analysis (intension-to-treat population)

Endpoints	Group	No.of patients	No. of patients with events	Stratified Hazard ratio	Cox regression	Stratified
					90%CI	log-rank test
DFS						*p* = 0.0549
	6M group	654	226	–	–	
	12M group	650	208	0.858	0.732–1.004	
RFS						*p* = 0.0143
	6M group	654	199	–	–	
	12M group	650	169	0.796	0.670–0.945	
OS						*p* = 0.0124
	6M group	654	113	–	–	
	12M group	650	87	0.727	0.575–0.919	

*DFS* disease-free survival, *RFS* relapse-free survival, *OS* overall survival

DFS Kaplan–Meier curve according to substage of stage III CRC was also shown in Supplementary Figure 2.

### Relapse-free survival

RFS was analysed based on 61.0 months of median follow-up with 368 events (199 and 169 events in the 6M and 12M groups, respectively). The 5-year RFS rate was 69.3% (95% CI: 65.57–72.69) and 74.1% (95% CI: 70.53–77.32) in the 6M and 12M groups, respectively. The HR was 0.796 (90% CI: 0.670–0.945; *p* = 0.0143). RFS in the 12M group was statistically superior to that of the 6M group (Fig. 2b, Table 2).

Recurrence was reported in 339 (26%) patients (6M group: 184 [28.1%]; 12M group: 155 [23.8%]). The most common site of recurrence in the 6M and 12M groups was the liver (76 (11.6%) and 59 (9.1%), respectively).

### Hazard rate of recurrence

**Fig. 3:**
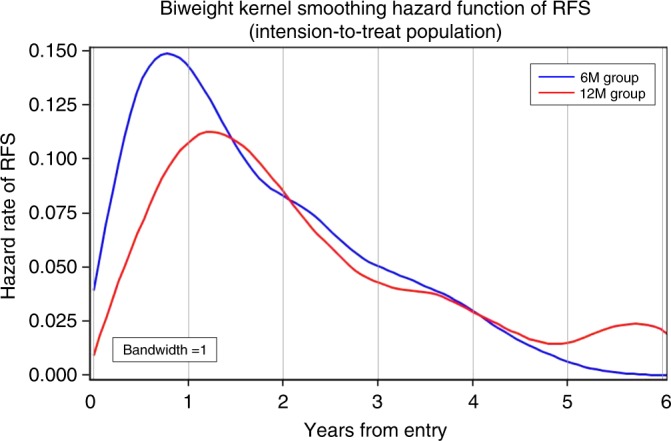
Biweight kernel smoothing hazard function. Relapse-free survival was exploratorily analysed as hazard of recurrence

The biweight kernel smoothing hazard function of RFS in the 6M and 12M groups is shown in Fig. 3. The hazard rate peaked at ~0.8 year and 1.2 year after registration in the 6M and 12M groups, respectively. Within 1.5 years, the hazard rate in the 6M group was higher than that of the 12M group; thereafter, they were similar. As regards DFS in the 6M group, the risk of events was evidently the highest during the first year after surgery, followed by a rapid reduction until ~approximately 5 years. The risk in the 12M group in 1 year was lower than that in the 6M group. The difference in the hazard rate between the two groups from year 0.5 to 1.5 was statistically significant (HR = 0.713; *p* = 0.0149).

### Overall survival

OS was analysed based on 63.3 months of median follow-up with 200 events (113 and 87 in the 6M group and 12M group, respectively). The 5-year OS was 83.2% (95% CI: 80.07–85.87) and 87.6% (95% CI: 84.73–89.89) in the 6M and 12M groups, respectively. The HR was 0.727 (90% CI: 0.575–0.919; *p* = 0.0124). The OS in the 12M group was statistically superior to that in the 6M group (Fig. 2c, Table 2).

### Subgroup analysis

**Fig. 4:**
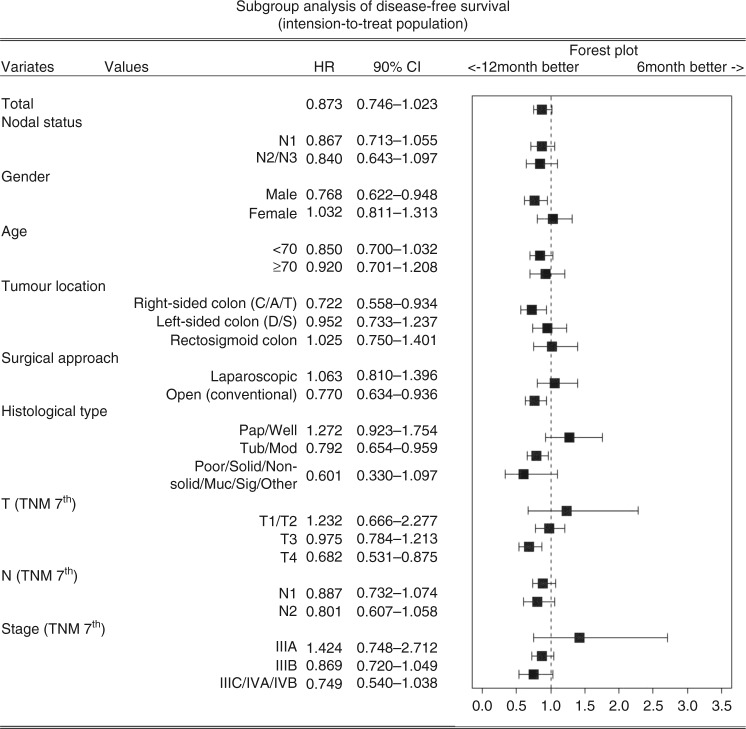
Subgroup analysis of disease-free survival (intension-to-treat population)

Unstratified subgroup analyses of DFS were performed for nodal status (N1, N2/N3), sex (male, female), age (<70, ≥70), tumour location (right-sided colon, left-sided colon, rectosigmoid colon), surgical approach (laparoscopic, open), histological type (papillary/well differentiated, tubular/moderately differentiated, poorly differentiated/mucinous/signet ring cell/other), tumour depth (T1/T2, T3, T4, according to the 7th TNM classification), lymph node metastasis (N1, N2, according to the 7th TNM classification), and TNM stage (IIIA, IIIB, IIIC/IVA/IVB, according to the 7th edition) (Fig. 4). The result showed a reduced risk of recurrence in the 12M group compared to the 6M group, particularly in the male sex, open surgical approach, and the T4 subgroup.

### Chemotherapy and safety

The completion rate which was defined as the ratio of patients who completed 6 or 12 months of capecitabine to the number of patients included in the safety analysis set of each treatment group was 71.5% and 46.1% in the 6M and 12M groups, respectively. Details of the safety analysis have been reported previously.^15^ The average total dose of capecitabine for 6M group was 1169 ± 409 tablets (mean ± SD) and that for 12M group was 1786 ± 910 tablets (mean ± SD). One tablet corresponds to 300 mg of capecitabine.

Briefly, the overall incidence rate of AEs was 91.7% and 94.7% in the 6M and 12M groups, respectively. The most common AE was hand-foot syndrome (HFS). Twelve months of adjuvant capecitabine demonstrated a higher cumulative incidence of HFS than the standard 6-month treatment; meanwhile, while toxicities even after 12-month capecitabine were clinically acceptable.

## Discussion

Recently, the final result of the JFMC33-0502 study was reported.^17^ It failed to show the superiority of 18 months adjuvant chemotherapy over 6 months regimen; however, because both stage IIB and III patients were included, and different drug intensities were used due to different administration schedule between the two arms in this study, and the results should be interpreted cautiously.

To the best of our knowledge, the present study is the first phase III trial on postoperative adjuvant chemotherapy in which the duration of oral 5-FU drug for CRC was directly compared. Primarily, 12 months capecitabine did not show superiority to 6 months regimen in terms of DFS. However, 12 months capecitabine showed significant superiority to 6 months both in RFS and OS. DFS events were defined as newly diagnosed cancer curable via local therapy. Our results show that adjuvant chemotherapy with capecitabine for 12 months has substantial clinical impact.

There are several possible explanations for the unmet primary result of this study. First, in the process of sample size estimation, we have assumed that 5-year DFS of 6M capecitabine would be 60% based on the result of the X-ACT study^5^ and that of 12M capecitabine would be 67%, which was based on our expectation without any solid evidence. Actually, the 5-year DFS rate was substantially higher than we assumed (6M: 65.3% vs 12M: 68.7%).

Second, the survival curves of two groups are not diverging over time but gradually getting closer. One possible explanation is that the longer administration of capecitabine might be effective especially in cases with a high risk of recurrence and just delay recurrence rather than cure.

Third, because we could have detected statistical difference in DFS when analysed both in all eligible cases and in all treated patients, these excluded ineligible and/or not-treated cases should have negative impacts on the difference in efficacy between 6M and 12M capecitabine.

Fourth, the statistical method used in this study might be inappropriate to analyse DFS. As shown in Fig. 2a, considering that the DFS curves became separated initially then later became closer, the log-rank test used in this study might not be necessarily suitable. Although not pre-planned, we also analysed DFS using stratified generalised Wilcoxon test and obtained statistically significant results (*p* = 0.0343 in all enrolled patients, *p* = 0.0151 in all eligible patients, and *p* = 0.0232 in all treated patients).

One limitation of this study was not adopting the 6 months oxaliplatin-based regimen, which is the current gold standard for adjuvant treatment of stage III CRC. This trial seems to deal with 2 non-standard therapies. However, oxaliplatin was not available as adjuvant treatment for CRC in Japan when this trial was planned. In addition, oral fluoropyrimidines alone has been used as adjuvant chemotherapy for long time and is adopted as one alternative regimen in the latest Japanese guideline.^18^

Recently, the result of IDEA, prospective, pre-planned, global pooled analysis of six randomised phase III trials, comparing the efficacy of 3 and 6 months FOLFOX/CAPOX in postoperative adjuvant chemotherapy for curatively resected stage III colon cancer has been reported.^19^ The non-inferiority of 3 months FOLFOX/Capeox to 6 months was not confirmed in the overall population. However, interestingly, in pre-planned subset analysis in CAPOX group, 3 months of therapy was shown to be as effective as 6 months. The main conclusion obtained from the subgroup analysis of IDEA trial was that 3 months treatment is effective enough for T1-3, N1 low risk stage III patients but not for T4 and/or N2 high risk stage III patients in which longer treatment, 6 months regimen, gave a better survival outcome. Then, we also performed an exploratory analysis on the DFS hazard ratio of 12M and 6M group in low-risk and high-risk subpopulations as same as the subgroup analysis performed in IDEA trial. We defined a low-risk group as patients with T1, T2, or T3 and N1 disease and a high-risk group as patients with T4, N2 or both as same as IDEA. Hazard ratio in low-risk group was 1.007 (95% CI: 0.753–1.345, *p* = 0.9650) and there was no difference in DFS between 6M and 12M capecitabine administration. However, hazard ratio in high-risk group was 0.746 (95% CI: 0.582–0.957, *p* = 0.0208). This result is consistent with the result shown in IDEA trial and suggest that longer treatment may be beneficial in high risk cases.

Then, how should we interpret the result in the present study. Our result suggesting more favourable outcome of longer administration of capecitabine seems to be against the current stream in this area. In regard to this point, our opinion is that we probably should consider the meaning and/or acting mechanism of post-operative adjuvant chemotherapy for CRC using oxaliplatin-containing regimen and oral 5 FU regimen separately. There might be the difference in the site and/or timing of recurrence between shorter administration of FOLFOX/CAPOX and longer administration of capecitabine alone. Further investigation is clearly needed.

Furthermore, Liu et al.^20^ showed that even patients with advanced cancer prefer oral chemotherapy to intravenous chemotherapy with the guarantee of equivalent efficacy. Of course, considering the poor tolerance of 2500 mg/m^2^ of capecitabine particularly reported in western elderly patients, an appropriate dose modification of capecitabine is very important in practice as described.^15^ In the future, the comparative study of prolonged administration of oral 5-FU monotherapy with 3 or 6 months oxaliplatin-based regimen would be of great interest, although such study is very hard to be conducted realistically.

Another important result drawn from this study is the good prognostic outcome in terms of DFS, RFS, or OS. As regards efficacy, the 5-year OS of the 12M group in the present study was high as 87.6%, while those in the global studies using oxaliplatin-based chemotherapy were 76.3% for FOLFOX4 in MOSAIC,^11^ 80.2% for FLOX in NSABP-C07,^12^ and 77.6% for XELOX in XELOXA.^13^ There are several possible biases for these results, including the difference in surgical or postoperative pathological procedures between countries. Recently, Shimada et al.^21^ reported that the optimal adjuvant chemotherapy should be chosen based on the risk of recurrence in each country, and Tsuji et al.^22^ also reported that the optimal adjuvant chemotherapy for CRC differed between Japanese and western strategies. In addition, the benefit of adjuvant chemotherapy in CRC itself should be re-evaluated in various aspects.^23^ Considering that 12 months capecitabine with better outcomes than 6 months is not associated with long-term neurotoxicity, this prolonged regimen might be considered as one alternative in the adjuvant setting for stage III CRC patients who refuse any additional toxicity of oxaliplatin or are not able to receive it.

In conclusion, the present study failed to show the superiority of 12 months capecitabine to 6 months regimen in terms of DFS. However, OS and RFS was statistically higher in the 12M group. Thus, the optimal duration of adjuvant chemotherapy, particularly for oral fluoropyrimidine, for stage III CRC needs further investigation. And 12 months of capecitabine monotherapy, along with 3 months of CAPOX with limited neurotoxicity, could be proposed as a treatment option without neurotoxicity.

## Supplementary information

Article File

Supplementary Figure 1

Supplementary Figure 2

Supplementary Table 1

JFMC37-0801, Study protocol

## Data Availability

All data and materials are available by inquiring to Japanese Foundation for Multidisciplinary Treatment of Cancer (JFMC).
