# Supplementary material for: Phase III randomised trial comparing 6 vs. 12-month of capecitabine as adjuvant chemotherapy for patients with stage III colon cancer: final results of the JFMC37-0801 study
Source: Br J Cancer. 2019 Mar 5;120(7):689–96. doi: 10.1038/s41416-019-0410-0 (PMC6461756; doi:10.1038/s41416-019-0410-0)
Supplement: Supplementary file 1 — Article File [file 41416_2019_410_MOESM1_ESM.docx]

**Supplementary material:**

Supplementary Figure 1. Study design of JFMC37-0801

Supplementary Figure 2. DFS Kaplan-Meier curve according to substage of stage III CRC

Supplementary Table 1. List of participating institutions (N=333)

Supplementary file. Study protocol of JFMC-37 trial
