# Supplementary figures and images for: Phase III randomised trial comparing 6 vs. 12-month of capecitabine as adjuvant chemotherapy for patients with stage III colon cancer: final results of the JFMC37-0801 study
Source: Br J Cancer. 2019 Mar 5;120(7):689–96. doi: 10.1038/s41416-019-0410-0 (PMC6461756; doi:10.1038/s41416-019-0410-0)

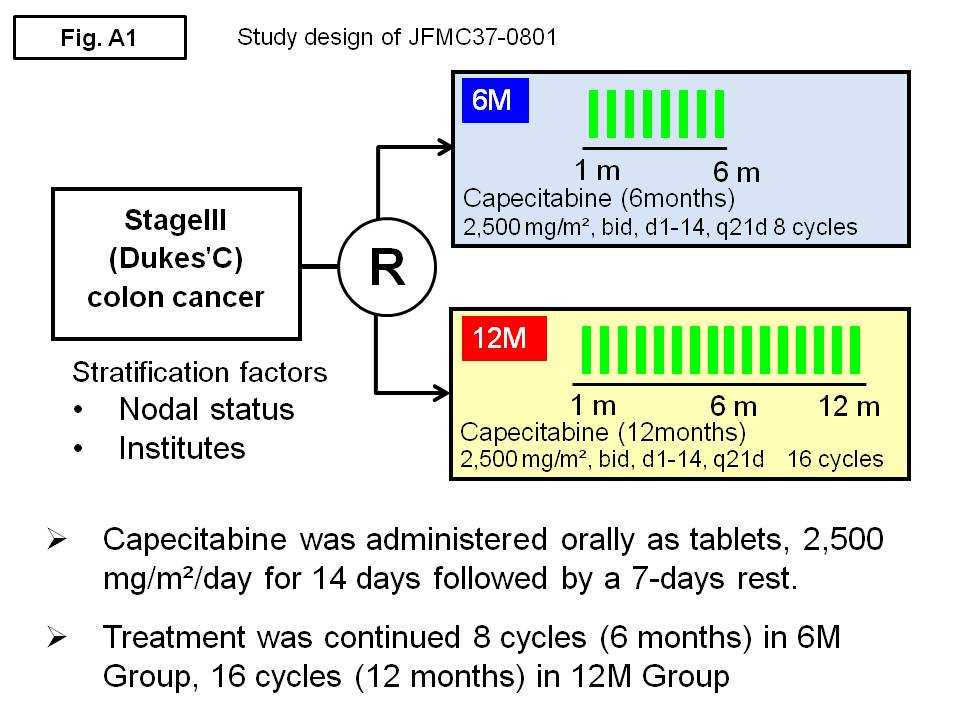

Supplement: Supplementary file 2 — Supplementary Figure 1 [file 41416_2019_410_MOESM2_ESM.tif]

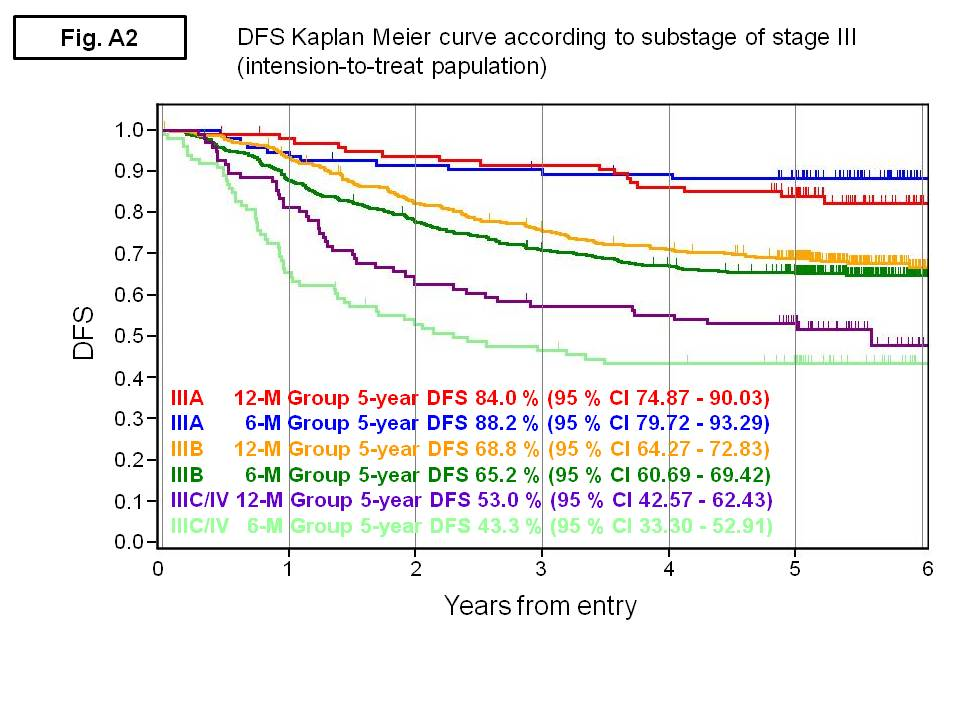

Supplement: Supplementary file 3 — Supplementary Figure 2 [file 41416_2019_410_MOESM3_ESM.tif]
