# Supplementary material for: Phase III randomised trial comparing 6 vs. 12-month of capecitabine as adjuvant chemotherapy for patients with stage III colon cancer: final results of the JFMC37-0801 study
Source: Br J Cancer. 2019 Mar 5;120(7):689–96. doi: 10.1038/s41416-019-0410-0 (PMC6461756; doi:10.1038/s41416-019-0410-0)
Supplement: Supplementary file 5 — JFMC37-0801, Study protocol [file 41416_2019_410_MOESM5_ESM.doc]

*JFMC37-0801*

**A Phase III, Randomized, Comparative Dose-Finding Study of Capecitabine as Postoperative Adjuvant Chemotherapy in Stage III (Dukes’ C) Colorectal Cancer Cases Involving Curative Resection**

**Clinical Study Protocol**

**Representative Investigator/Protocol Submitted by:**

**Naohiro Tomita, Hyogo College of Medicine**

**Japanese Foundation for Multidisciplinary Treatment of Cancer**

ver.1.1　06/18/2008

ver.1　06/14/2008

**Table of Contents**

[0. Study Overview 5](#__RefHeading___Toc201563455)

[0-1. Schema 5](#__RefHeading___Toc201563456)

[0-2. Study Objectives 5](#__RefHeading___Toc201563457)

[0-3. Subjects Targeted 5](#__RefHeading___Toc201563458)

[0-4. Treatment Procedure 6](#__RefHeading___Toc201563459)

[0-5. Study Period and Target Number of Subjects 7](#__RefHeading___Toc201563460)

[0-6. Study Organization 7](#__RefHeading___Toc201563461)

[1. Objectives 9](#__RefHeading___Toc201563462)

[2. Study Background 9](#__RefHeading___Toc201563463)

[2-1. Overview of Target Disease 9](#__RefHeading___Toc201563464)

[2-2. Postoperative Adjuvant Chemotherapy for Colorectal Cancer 9](#__RefHeading___Toc201563465)

[2-3. Profile of Capacitabine 12](#__RefHeading___Toc201563466)

[3. Selection Criteria 14](#__RefHeading___Toc201563467)

[3-1. Inclusion Criteria 14](#__RefHeading___Toc201563468)

[3-2. Exclusion Criteria 14](#__RefHeading___Toc201563469)

[4. Subject Enrollment and Group Assignment 15](#__RefHeading___Toc201563470)

[4-1. Study Design 16](#__RefHeading___Toc201563471)

[4-2. Subject Enrollment 16](#__RefHeading___Toc201563472)

[4-3. Group Randomization 16](#__RefHeading___Toc201563473)

[5. Treatment Procedures 17](#__RefHeading___Toc201563474)

[5-1. Treatment Protocol 17](#__RefHeading___Toc201563475)

[5-2. Study Drug 17](#__RefHeading___Toc201563476)

[5-3. Dosing Schedule 17](#__RefHeading___Toc201563477)

[5-4. Treatment Adjustment Provisions 18](#__RefHeading___Toc201563478)

[5-5. Treatment Discontinuation Provisions 20](#__RefHeading___Toc201563479)

[5-6. Safety Concerns 21](#__RefHeading___Toc201563480)

[5-7. Concomitant Treatments 22](#__RefHeading___Toc201563481)

[5-8. Post-treatment 24](#__RefHeading___Toc201563482)

[6. Examination/Observation Items 25](#__RefHeading___Toc201563483)

[6-1. Pre-Enrollment Examination/Observation Items 25](#__RefHeading___Toc201563484)

[6-2. Examination/Observation Items During Treatment Protocol and Observation Periods 25](#__RefHeading___Toc201563485)

[6-3. Checking Procedures for Recurrent and Non-Recurrent Cancerous Lesions 26](#__RefHeading___Toc201563486)

[6-4. Outcome Investigation 26](#__RefHeading___Toc201563487)

[6-5. Discontinuation of Treatment Protocol 26](#__RefHeading___Toc201563488)

[7. Study Endpoints 28](#__RefHeading___Toc201563489)

[7-1. Primary Endpoint 28](#__RefHeading___Toc201563490)

[7-2. Secondary Endpoints 28](#__RefHeading___Toc201563491)

[8. Statistical Matters (Evaluation Criteria and Statistical Analyses) 30](#__RefHeading___Toc201563492)

[8-1. Definition of the Target Population for Analysis 30](#__RefHeading___Toc201563493)

[8-2. Primary Endpoint Analyses 30](#__RefHeading___Toc201563494)

[8-3. Secondary Endpoint Analyses 30](#__RefHeading___Toc201563495)

[8-4. Target Number of Subjects and Statistical Basis 30](#__RefHeading___Toc201563496)

[8-5. Revision to the Number of Subjects 31](#__RefHeading___Toc201563497)

[8-6. Study Period 32](#__RefHeading___Toc201563498)

[8-7. Interim Analysis and Early Study Termination 32](#__RefHeading___Toc201563499)

[9. Emergency Reporting of Adverse Events 32](#__RefHeading___Toc201563500)

[9-1. Events Warranting Emergency Reporting 33](#__RefHeading___Toc201563501)

[9-2. Reporting Procedure 33](#__RefHeading___Toc201563502)

[9-3. Management by the Study Secretariat 33](#__RefHeading___Toc201563503)

[10. Data Collection and Retention 34](#__RefHeading___Toc201563504)

[10-1. Submission of Case Report Forms, Etc. 34](#__RefHeading___Toc201563505)

[10-2. Recordkeeping and Retention Provisions 34](#__RefHeading___Toc201563506)

[11. Ethical Matters 34](#__RefHeading___Toc201563507)

[11-1. Compliance Requirements 34](#__RefHeading___Toc201563508)

[11-2. Informed Consent 34](#__RefHeading___Toc201563509)

[11-3. Retention of Subjects’ Personal Information and Subject Identifiability 35](#__RefHeading___Toc201563510)

[11-4. Protocol Adherence 35](#__RefHeading___Toc201563511)

[11-5. Study Site Institutional Review Board (IRB) Approval 35](#__RefHeading___Toc201563512)

[12. Study-related Expenses and Compensation 35](#__RefHeading___Toc201563513)

[12-1. Funding Sources and Financial Relationships 35](#__RefHeading___Toc201563514)

[12-2. Study-related Expenses 35](#__RefHeading___Toc201563515)

[12-3. Injury-related Compensation 35](#__RefHeading___Toc201563516)

[13. Clinical Study Protocol Amendments 35](#__RefHeading___Toc201563517)

[14. Monitoring 36](#__RefHeading___Toc201563518)

[15. Publication of Study Outcomes 36](#__RefHeading___Toc201563519)

[16. Study Organization 36](#__RefHeading___Toc201563520)

[16-1. Representative Investigator/Protocol Submitter 36](#__RefHeading___Toc201563521)

[16-2. Executive Committee 37](#__RefHeading___Toc201563522)

[16-3. Principal Investigator 38](#__RefHeading___Toc201563523)

[16-4. Data and Safety Monitoring Committee 38](#__RefHeading___Toc201563524)

[16-5. Supervising Statistician 38](#__RefHeading___Toc201563525)

[16-6. HRQOL/Medical Economics Review Committee 38](#__RefHeading___Toc201563526)

[16-7. Participating Study Sites 38](#__RefHeading___Toc201563527)

[16-8. Study Secretariat 38](#__RefHeading___Toc201563528)

[17. References 39](#__RefHeading___Toc201563529)

**Appendix 1 List of participating study sites for JFMC33-0502**

**APPENDIX 2 Patient briefing materials/Informed Consent Form**

**ATTACHMENTS Case report forms**

# **0. Study Overview**

## **0-1. Schema**

Stage III (Dukes’ C)

Colorectal Cancer, Sigmoid Colon Cancer Cases

Curative Resection “Cur A”

Group Randomization

**Group A: Standard-of-Care**

Capecitabine 2 weeks on, 1 week off

8 courses (approx. 6 months)

**Group B: Study Treatment**

Capecitabine 2 weeks on, 1 week off

16 courses (approx. 12 months)

## **0-2. Study Objectives**

The purpose of this study is to determine the optimal dosing period of capecitabine as postoperative adjuvant chemotherapy in cases of Stage III (Dukes’ C) colorectal cancer and sigmoid colon cancer involving curative resection by comparing patients receiving 8 courses (approximately 6 months) of the standard-of-care therapy consisting of capecitabine administered in a 2 weeks on, 1 week off schedule (Group A) with patients receiving 16 courses of capecitabine (approximately 12 months) on the same dosing schedule (Group B).

Primary Endpoint:

Disease-free survival (DFS) period

Secondary Endpoints:

(1) Relapse-free survival (RFS) period

(2) Overall survival (OS) period

(3) 2-year DFS rate

(4) Adverse event frequency and severity

(5) Treatment compliance

(6) HRQOL (Health-related Quality of Life)/Medical Economy　*Tertiary study conducted by capable study sites

## **0-3. Cases Targeted**

**0-3-1. Inclusion Criteria**

1) Patients with histologically diagnosed colon cancer (adenocarcinoma)

2) Cases of histological Stage III* colorectal cancer or sigmoid rectal cancer

*as defined in the Treatment Guidelines for Colorectal Cancer (7th edition)

3) Colorectal cancer cases in which resective surgery was performed (including cases involving D2 or higher lymph node dissection)

4) Cases in which surgery was determined to have achieved cure degree A (Cur A)

5) Patients aged (at time of enrollment) between 20 and 80 years

6) Performance status (PS) (Eastern Cooperative Oncology Group [ECOG]): 0 or 1

7) Eligible patients who have not yet undergone chemotherapy or radiotherapy

8) Patients capable of taking meals

9) Patients maintaining stable vital organ functions

a) WBC count ≥ 3,000/mm^3^ and <12,000/mm^3^

b) Hemoglobin ≥ 9.0 g/dL

c) Thrombocyte count ≥ 90,000/mm^3^

d) Serum creatinine ≤ 1.5x hospital standard upper limit

e) Total serum bilirubin ≤ 1.5x hospital standard upper limit

f) AST, ALT ≤ 2.5x hospital standard upper limit

g) ALP ≤ 2.5x hospital standard upper limit

10) Creatinine clearance* >50 mL/min

*: Determined based on the following simple formula to calculate creatinine clearance

Men: [(140-age)×body weight (kg)]/[72×serum creatinine value (mg/dL)]

Women: [(140-age)×body weight (kg)×0.85]/[72×serum creatinine value (mg/dL)]

11) Cases in which the protocol treatment was initiated within 8 weeks after surgery

12) Patients who consented to study participation in writing

**0-3-2. Exclusion Criteria**

1) Patients who are pregnant or nursing, or who may become pregnant

2) Patients who have a history of hypersensitivity or who have developed severe adverse events as a result of fluoropyrimidine drug use

3) Patients who have undergone organ transplantation

4) Patients who have serious complicating conditions (interstitial pneumonia, pulmonary fibrosis, intestinal palsy, intestinal obstruction, poorly managed diabetes, cirrhosis, poorly managed hypertension, history of myocardial infarction within the prior 6 months, unstable angina, etc.)

5) Patients with multiple active* (*defined as cases with a disease-free period shorter than 5 years) cancers (excluding resolved skin basal cell carcinoma and cervical cancer, or gastric, esophageal, and M colon cancers confirmed to have been resolved as a result of endoscopic mucosal resection)

*“Multiple cancer” is a blanket term encompassing both “multifocal” and “duplicative” cancers [Treatment Guidelines for Colorectal Cancer (7th edition)]

6) Patients with uncontrollable advanced complicating infection

7) Patients otherwise deemed unsuitable for participation in this clinical trial by a physician

## **0-4. Treatment Procedure**

**0-4-1. Group A: Standard-of-care group (8 courses [approx. 6 months])**

Capecitabine (Xeloda tablets): Patients will receive the established dosage of capecitabine (1,250 mg/m^2^) based on their total body surface area (1,500 mg–2,400 mg) twice daily (after breakfast and within 30 minutes after dinner) for 14 days continuously followed by a 7-day off period. This 3-week period will comprise 1 course of treatment. Patients will receive 8 courses in total.

After completing all 8 courses, patients not exhibiting cancerous recurrence will undergo continuous monitoring without additional treatment.

**0-4-2. Group B: Study treatment group (16 courses [approx. 12 months])**

Capecitabine (Xeloda tablets): Patients will receive the established dosage of capecitabine (1,250 mg/m^2^) based on their total body surface area (1,500 mg–2,400 mg) twice daily (after breakfast and within 30 minutes after dinner) for 14 days continuously followed by a 7-day off period. This 3-week period will comprise 1 course of treatment. Patients will receive 16 courses in total.

After completing all 16 courses, patients not exhibiting cancerous recurrence will undergo continuous monitoring without additional treatment.

**0-4-3. Established Capecitabine Dosages**

Per-administration capecitabine dosages

by subject body surface area (Groups A and B)

| Body Surface Area | Single-dose Volume |
| --- | --- |
| ≤1.33 m^2^ | 1,500 mg (5 tablets) |
| ≥1.33 m^2^ ≤1.57 m^2^ | 1,800 mg (6 tablets) |
| ≥1.57m^2^ ≤1.81m^2^ | 2,100mg (7 tablets) |
| ≥1.81m^2^ | 2,400mg (8 tablets) |

## **0-5. Study Period and Target Number of Subjects**

Target number of subjects: 600 subjects per group, 1200 in total

Enrollment period: 2 years (Sept. 2008–Aug. 2010)

Follow-up period: 5 years after last enrollment

Cumulative study period: 7 years (Sept. 2008–Aug. 2015)

## **0-6. Study Organization**

Principal Investigator/Protocol Coordinator:

Naohiro Tomita, Department of Surgery, Hyogo College of Medicine

Research Management Investigators:

Sadahiro Sotaro, Department of Gastrointestinal Surgery, Tokai University

Hideyuki Mishima, Department of Surgery, NHO Osaka National Hospital

Participating Study Sites: ~300 sites (APPENDIX 1)

Study Secretariat: Japanese Foundation for Multidisciplinary Treatment of Cancer (JFMC)

Tani Bldg., 3F, 1-28-6 Kameido, Koto-ku, Tokyo, Japan 136-0071

TEL: 03-3239-2011 (data center)

03-3239-2341 (study representative/administration)

0120-184100 (toll-free)

FAX: 03-3239-2553

E-mail: jfmc-dc@jfmc.or.jp (data center)

jfmc@jfmc.or.jp (study representative/administration)

Please use the following contact information until September 12, 2008 (no changes to TEL, FAX, or E-mail information):

Chiyoda K Bldg., 4F, Kudan-minami 3-7-14, Chiyoda-ku, Tokyo, Japan 102-0074

# **1. Study Objectives**

The purpose of this study is to determine the optimal dosing period of capecitabine as postoperative adjuvant chemotherapy in cases of Stage III (Dukes’ C) colorectal cancer and sigmoid colon cancer involving curative resection by comparing patients receiving 8 courses (approximately 6 months) of the standard-of-care therapy consisting of capecitabine administered in a 2 weeks on, 1 week off schedule (Group A) with patients receiving 16 courses of capecitabine (approximately 12 months) on the same dosing schedule (Group B).

Primary Endpoint:

Disease-free survival (DFS) period

Secondary Endpoints:

(1) Relapse-free survival (RFS) period

(2) Overall survival (OS) period

(3) 2-year DFS rate

(4) Adverse event frequency and severity

(5) Treatment compliance

(6) HRQOL (Health-related Quality of Life)/Medical Economy　*Tertiary study conducted by capable study sites

# **2. Study Background**

## **2-1. Overview of Target Disease**

**2-1-1. Colon Cancer Epidemiology**

The number of colon cancer cases in Japan increases every year. This figure was estimated at 60,732 cases in 1990, and rose sharply to approximately 100,137 cases in 2001 (a prevalence of approximately 78.7 cases per 100,000 people). In addition, colon cancer has become the second most common type of cancer in men, after gastric cancer, and the most common type of cancer in women. In terms of mortality frequency, 24,778 people died from colon cancer in Japan in 1990, and this figure rose to 41,097 people by 2005 (a mortality rate of approximately 32.7 deaths per 100,000 people). Colon cancer has become the fourth most common form of cancer in men (preceded by lung, gastric, and liver cancers), and the most common form of cancer in women ^1)^.

**2-1-2. Colorectal cancer prognosis**

The first-line treatment option for colorectal cancer is surgery, but according to a national survey conducted by the Japanese Society for Cancer of the Colon and Rectum (JSCCR), the 5-year survival rate by stage classification in colon cancer treated with radical surgery is 90.4% for Stage I, 82.0% for Stage II, 69.2% for Stage III, and 15.9% for Stage IV. Meanwhile, the 5-year survival rate by stage following surgery for rectal cancer is 91.4% for Stage I, 76.4% for Stage II, 57.5% for Stage III, and 14.6% for Stage IV ^2)^. As such, although the respective 5-year survival rates for Stage I and Stage II cases are relatively favorable, the recurrence rate for Stage III cases is high, and adjuvant chemotherapy administered with the aim of suppressing postoperative cancerous recurrence is necessary in the treatment of colorectal cancer. The appropriate utilization of postoperative adjuvant chemotherapy is believed to ultimately contribute to improved survival prognosis in cases of colorectal cancer.

## **2-2. Postoperative adjuvant therapy for colon cancer**

**2-2-1. Postoperative adjuvant therapy for colon cancer**

In Europe and the United States, the results of the Intergroup, NSABP, and IMPACT studies conducted primarily by the North Central Cancer Treatment Group (NCCTG) in the 1990s demonstrated the clinical utility of postoperative adjuvant chemotherapy surgery alone in Stage III colorectal cancer cases. Additionally, 5-FU + LEV chemotherapy was designated as a standard treatment for Stage III colorectal cancer at the National Institutes of Health (NIH) consensus conference in 1990. 5-FU + LEV chemotherapy was subsequently established as the control arm for this comparative study. Results of subsequent clinical studies, such as the INT0089 study, suggested there was little benefit in adding LEV to 5-FU + LV therapy, that 5-FU + high-dose LV and 5-FU + low-dose LV demonstrated similar performance, and that 5-day continuous administration and once-weekly administration of LV also achieved similar performance ^3)-7)^. Based on these results, 5-FU/LV therapy was established as the standard-of-care for postoperative adjuvant chemotherapy for Stage III colorectal cancer cases. In addition, studies investigating oral 5-FU were also conducted. In the NSABP C-06 study investigating UFT + LV therapy in Stage II/III colorectal cancer cases, UFT + LV exhibited noninferiority compared with 5-FU/LV (disease-free survival [DFS]: hazard ratio [HR] 1.004, overall survival [OS]: HR 1.014) ^8)^. In the X-ACT study of capecitabine for Stage III colorectal cancer, not only was noninferiority of capecitabine monotherapy (DFS: HR 0.86, OS: HR 0.84) demonstrated in comparison with 5-FU/LV therapy, but significant trends were also observed during the superiority study, and significant improvement was observed with respect to relapse-free survival (RFS) ^9)^. Combination therapies containing oxaliplatin and irinotecan hydrochloride were also evaluated, and significant improvement in DFS was not observed with the use of irinotecan hydrochloride in the CALGB C89803, ACCORD-02, or PETACC-3 studies ^10)-12)^. Meanwhile, oxaliplatin demonstrated significant improvements in DFS in the MOSAIC and NSABP C-07 studies ^13), 14)^.

Many Japanese studies of oral 5-FU drugs have also been conducted. Individual tests of a surgery only patient group have failed to demonstrate the superiority of oral 5-FU drugs, but the results of a meta-analysis of the No. 7 and No. 15 studies conducted by the Japanese Foundation for Multidisciplinary Treatment of Cancer (JFMC) revealed that 1 year of oral 5-FU drug therapy was significantly superior in terms of DFS and OS in Stage III colorectal cancer cases ^15)^. Randomized controlled trials (RCTs) investigating 5-FU/LV therapy and UFT + LV therapy for Stage III colorectal cancer are currently underway in Japan.

**2-2-2. Standard postoperative adjuvant chemotherapy regimens for colorectal cancer recommended by Japanese and overseas guidelines**

The following treatment regimens are recommended in the Japanese “JSCCR Guidelines 2005 for the Treatment of Colorectal Cancer” and the American National Comprehensive Cancer Network (NCCN) Clinical Practice Guidelines in Oncology 2008 (NCCN Guidelines®) v.1.

Table 1 Standard postoperative adjuvant chemotherapy regimens for colorectal cancer recommended by Japanese and overseas treatment guidelines

| Guideline | Regimen | No. of courses/dosing period |
| --- | --- | --- |
| Guidelines for the treatment of colon cancer | 5-FU/LV therapy (RPMI regimen: bolus) | 6 months |
| NCCN  Guidelines* | 5-FU/LV(RPMI regimen: bolus) | 4 courses (8 months) |
|  | 5-FU/LV(Machover regimen: bolus) | 6 courses (6–7.5 months) |
|  | Capecitabine (2,500 mg/m^2^ twice daily on days 1–14 (repeat every 3 weeks) | 8 courses (6 months) |
|  | 5-FU/LV + oxaliplatin(FOLFOX4 regimen: infusion) | 12 courses (6 months) |
|  | 5-FU/LV + oxaliplatin(mFOLFOX6 regimen infusion) | 12 courses (6 months) |

* The National Comprehensive Cancer Network (NCCN) Clinical Practice Guidelines in Oncology (NCCN Guidelines®) concern Category 2A or higher

**2-2-3. Period of postoperative adjuvant chemotherapy for colon cancer**

Drug selection and administration period with respect to postoperative adjuvant chemotherapy for colorectal cancer were evaluated in the following 3 studies. In the INT-0089 study targeting high-risk Stage II and Stage III colorectal cancer cases, 5-FU/LEV (12 months) was administered to the control group, and 5-FU/LV (low-dose LV: 6 months) and 5-FU/LV (high-dose LV: 8 months), and 5-FU/LV+LEV (low-dose LV: 6 months) were administered to the experimental groups. Comparison with respect to DFS and OS revealed no significant differences between the groups, and it was determined that 5-FU/LV (6 months) is preferable to 5-FU/LEV (12 months) in terms of toxicity, cost, and patient burden ^3)^. The NCCTG 894651 study also compared 4 groups of high-risk Stage II and Stage III colorectal cancer cases receiving 6-month or 12-month administration of 5-FU/LEV. Six-month administration of 5-FU/LEV/LV demonstrated a significantly better 5-year survival rate compared with 6-month 5-FU/LEV, but no significant difference was observed between 12-month 5-FU/LEV and 12-month 5-FU/LEV/LV. Moreover, in the NSABP C-04 study evaluating the efficacy of 5-FU/LV (once weekly), in comparison to 12-month 5-FU/LEV and 5-FU/LEV/LV as treatment for Stage II/III colorectal cancer, once weekly 5-FU/LV demonstrated significantly better DFS when compared with 12-month 5-FU/LEV, and no significant difference when compared to 12-month 5-FU/LEV/LV ^16)^.

Based on the above observations, 6 months was determined to be the standard treatment period for postoperative adjuvant chemotherapy for colorectal cancer.

In addition, because capecitabine and UFT-LV in the X-ACT and NSABPC-06 studies, respectively, were administered over 6-month periods, 6 months has also become the standard administration period for oral 5-FU drugs administered as part of postoperative adjuvant chemotherapy regimens for colorectal cancer.

However, because the test results forming the basis for this designation did not have sufficient detection sensitivity during their respective treatment periods, and because there were differences in the treatment periods among these studies, the National Cancer Institute Physician Data Query (NCI PDQ) states that the current evidence supporting this treatment period is not definitive.

In addition, the results of a meta-analysis indicated that 1-year administration of oral 5-FU drugs in Stage III colorectal cancer cases achieved significantly superior DFS and OS compared to the surgery only group ^15)^, and in numerous Japanese clinical trials, the period of administration for oral 5-FU drugs is often set as 1 year or longer, so it cannot be concluded that the optimal treatment period for oral 5-FU drugs as adjuvant postoperative chemotherapy for colorectal cancer is 6 months. Currently, the JFMC33-0502 (No. 33) study conducted by the JFMC is evaluating daily UFT/LV (6 months) in comparison to UFT/LV administered in 5 doses over 2 days (18 months).

**2-2-4. Relationship between treatment period and time to cancerous recurrence in postoperative adjuvant chemotherapy for colorectal cancer**

The hazard function was estimated using the colorectal cancer-related data from JFMC studies No. 7 and No. 15 as well as the X-ACT study data, and was used to assess the timing and risk of cancerous recurrence.

Changes in the risk of recurrence between the surgery only group and the 1-year oral 5-FU drug therapy (UFT, HCFU) group are shown in Fig. 1. Changes in the recurrence risk in the 6-month 5-FU/LV group and the 6-month capecitabine monotherapy group during the X-ACT study are shown in Fig. 2.

The recurrence risk in the surgery only group, 6-month 5-FU/LV, and 6-month capecitabine monotherapy groups peaked between 1 and 2 years postoperatively. However, no peak recurrence risk was observed in the 1-year oral 5-FU drug therapy (UFT, HCFU) group.

Fig. 1 JFMC No. 7 and No. 15 studies: differences in recurrence risk between Stage II/III colorectal cancer patient groups receiving surgery only or oral 5-FU drug therapy for 1 year

DFS

Surgery-only group　　　　　　　1 year of oral 5-FU drug group

Fig. 2 X-ACT study: differences in recurrence risk between Stage III colorectal cancer patient groups receiving 5-FU/LV therapy for 6 months or capecitabine monotherapy for 6 months

DFS

## **2-3. Profile of Capecitabine**

**2-3-1. Overseas clinical trials investigating postoperative capecitabine monotherapy for colon cancer**

In early 1998, oral capecitabine (1,250 mg/m^2^, twice daily for 21 days, administered continuously on days 1–14, off-drug for the final 7 days) was administered postoperatively to 1956 patients with Dukes’ Stage C colorectal cancer via rapid intravenous injection as part of a randomized Phase III comparative study against 5-FU/LV (Mayo Clinic regimen) (6 month treatment period for each group). In the safety analysis group (993 patients in the capecitabine treatment group, 974 patients in the rapid IV 5-FU/LV group), patients in the capecitabine group exhibited significantly lower incidences of diarrhea, stomatitis, nausea/vomiting, and neutropenia in comparison to the 5-FU/LV group (*p*<0.001), but higher incidence of hand-foot syndrome. The number of patients who developed Grade III or IV neutropenia, febrile neutropenia/sepsis, or stomatitis was lower in the capecitabine group compared with the 5-FU/LV group (*p*<0.001). However, the number of patients who developed Grade III hand-foot syndrome was higher in the capecitabine group (*p*<0.001). The safety profile of capecitabine was similar in patients under and over 65 years of age ^17)^. Regarding DFS, the primary endpoint of this study, capecitabine was demonstrated to have achieved equal or superior results compared with rapid IV 5-FU/LV (Mayo Clinic regimen) (HR 0.87 [95% confidence interval (CI): 0.75-1.00]) ^18)^.

**2-3-2. Overseas clinical trials investigating postoperative capecitabine monotherapy for metastatic colon cancer**

Two multicenter, open-label Phase III clinical studies were jointly conducted to compare intermittent administration of capecitabine against rapid intravenous 5-FU/LV (Mayo Clinic regimen, the standard regimen designated by regulations at the time) as first-line treatment for metastatic colorectal cancer ^19, 20)^. The same protocol was used for these studies, and an integrated analysis of all data was prospectively designed. A total of 1207 patients received oral capecitabine (1,250 mg/m^2^, twice daily for 21 days, given continuously for days 1–14, off-drug for the final 7 days; n=603) or rapid intravenous 5-FU/LV (Mayo Clinic regimen; n=604). Patients who received capecitabine exhibited significantly better response rates compared with the 5-FU/LV group (26% vs. 17%; *p*<0.0002). The median time to response, time to effect loss, and duration of response were all similar across the groups. In addition, the times to disease progression (TTP) were comparable between the groups (capecitabine and 5-FU/LV, median period: 4.6 and 4.7 months, respectively). The survival periods were also comparable (median periods: 12.9 and 12.8 months, respectively). A subgroup analysis revealed that capecitabine administration consistently resulted in superior response rates (*p*<0.05) even in the patient subgroup with poor prognostic indicators. Multivariate Cox regression analysis confirmed that poor performance status (PS) (Karnofsky Performance Status [KPS] ≤ 80 vs 100), liver as the primary site of metastasis, and multiple metastases vs. single metastasis were independent prognostic indicators of low survival rate ^21)^.

Based on these results, capecitabine was approved by various regulators worldwide as a first-line monotherapy for patients with metastatic colorectal cancer.

**2-3-3. Overseas Phase II clinical trials targeting nonresectable colon cancer**

In an overseas study investigating the safety and efficacy of capecitabine, 1,250 mg/m^2^ of capecitabine was orally administered twice daily for 21 days continuously with a 7-day off-period to 60 patients with nonresectable colorectal cancer who were chemotherapy-naïve or who had completed preoperative or postoperative adjuvant chemotherapy 6 months prior. A response rate of 35.0% was observed, and major adverse events included hand-foot syndrome in 73.3% of patients, skin discoloration in 38.3%, diarrhea in 35.0%, nausea in 35.0%, stomatitis in 35.0%, and diminished appetite in 33.3% of patients ^22)^.

# **3. Selection Criteria**

## **3-1. Inclusion Criteria**

1) Patients histologically diagnosed with colon cancer (adenocarcinoma)

2) Cases of histological Stage III* colorectal cancer or sigmoid rectal cancer

*as defined in the Treatment Guidelines for Colorectal Cancer (7th edition)

3) Colorectal cancer cases in which resective surgery was performed (including cases involving D2 or higher lymph node dissection)

4) Cases in which surgery was determined to have achieved cure degree A (Cur A)

5) Patients aged (at time of enrollment) between 20 and 80 years

6) Performacne Status (PS) (Eastern Cooperative Oncology Group [ECOG]): 0 or 1

7) Eligible patients who have not yet undergone chemotherapy or radiotherapy

8) Patients capable of taking meals

9) Patients maintaining stable vital organ functions

a) WBC count ≥ 3,000/mm^3^ and <12,000/mm^3^

b) Hemoglobin ≥ 9.0 g/dL

c) Thrombocyte count ≥ 90,000/mm^3^

d) Serum creatinine ≤ 1.5x hospital standard upper limit

e) Total serum bilirubin ≤ 1.5x hospital standard upper limit

f) AST, ALT ≤ 2.5x hospital standard upper limit

g) ALP ≤ 2.5x hospital standard upper limit

10) Creatinine clearance* >50 mL/min

*: Determined based on the following simple formula to calculate creatinine clearance

Men: [(140-age)×body weight (kg)]/[72×serum creatinine value (mg/dL)]

Women: [(140-age)×body weight (kg)×0.85]/[72×serum creatinine value (mg/dL)]

11) Cases in which the protocol treatment was initiated within 8 weeks after surgery

12) Patients who consented to study participation in writing

## **3-2. Exclusion Criteria**

1) Patients who are pregnant or nursing, or who may become pregnant

2) Patients who have a history of hypersensitivity or have developed severe adverse events as a result of fluoropyrimidine drug use

3) Patients who have undergone organ transplantation

4) Patients with serious complicating conditions (interstitial pneumonia, pulmonary fibrosis, intestinal palsy, intestinal obstruction, poorly managed diabetes, cirrhosis, poorly managed hypertension, history of myocardial infarction within the prior 6 months, unstable angina, etc.)

5) Patients with multiple active* (*defined as cases with a disease-free period shorter than 5 years) cancers (excluding resolved skin basal cell carcinoma and cervical cancer, or gastric, esophageal, and M colon cancers confirmed to have been resolved as a result of endoscopic mucosal resection.)

*“Multiple cancer” is a blanket term encompassing both “multifocal” and “duplicative” cancers (Treatment Guidelines for Colorectal Cancer [7th edition])

6) Patients with uncontrollable advanced complicating infections

7) Patients otherwise deemed unsuitable for participation in this clinical trial by a physician

# **4. Subject Enrollment and Group Assignment**

## **4-1. Study Design**

Stage III (Dukes’ C)

Colorectal Cancer, Sigmoid Colon Cancer Cases

Curative Resection “Cur A”

Group Randomization

**Group A: Standard-of-Care**

Capecitabine 2 weeks on, 1 week off

8 courses (approx. 6 months)

**Group B: Study Treatment**

Capecitabine 2 weeks on, 1 week off

16 courses (approx. 12 months)

## **4-2. Subject Enrollment**

This study utilized centralized subject enrollment.

1) Register so that treatment can be started within 8 weeks after the date of surgery after confirmation of eligibility under “3. Selection Criteria”. (Note: enroll after confirmation of pathological test results)

2) After obtaining patient consent to study participation, fill out the "case registration form" (Form 1-1) and send to the secretariat via fax.

3) After receiving the case registration form via fax, the secretariat confirms that the prospective subject meets the selection criteria, then calls the study site supervisor as soon as possible. Subject group assignment then occurs, and dosing based on the subject total body surface area is communicated to the site by the secretariat, completing subject “enrollment”. The study secretariat then promptly sends the case registration confirmation form (Form 1-2) to the study site. Each site retains the case registration confirmation form on site and manages subject identification by creating a subject registry, etc.

4) The protocol treatment is initiated within 14 days after enrollment.

* “As soon as possible” means for subject enrollment (9:00-17:00) during business hours (Monday-Friday, excluding public holidays and the Year-End holiday period [December 29-January 4]), within “30 minutes” when submitted via paper during business hours (9:00-17:00) or between 9:00-9:30 the following business day if submitted outside of business hours (e.g., 9:00-9:30 on the next working day).

Japanese Foundation for Multidisciplinary Treatment of Cancer

Tani Bldg., 3F, 1-28-6 Kameido, Koto-ku, Tokyo, Japan 136-0071

TEL: 03-3239-2011 (data center)

03-3239-2341 (study representative/administration)

0120-184100 (toll-free)

FAX: 03-3239-2553

E-mail: jfmc-dc@jfmc.or.jp (data center)

Hours: Monday-Friday, 9:00-17:00 (excluding weekends, national holidays, and the New Year period (12/29–1/4))

## **4-3. Group Randomization**

The following factors are designated as adjustment factors to minimize bias during subject randomization.

1) N-factor (N1/N2, N3)

2) Study site

**5. Treatment Procedures**

## **5-1. Protocol Treatment**

The protocol treatment assigned to each subject starts within 8 weeks after surgery. Subjects’ height and body weight at enrollment are used to calculate dosage, and no dose correction due to changes in body weight is performed.

## **5-2. Study Drug**

Capecitabine

- Xeloda® tablets 300 (Chugai Pharmaceutical Co., Ltd.)

1 tablet contains 300 mg of capecitabine

## **5-3. Dosing Schedule**

**5-3-1. Group A: Standard-of-care group (8 courses [approx. 6 months])**

Capecitabine (Xeloda tablets): Patients will receive the established dosage of capecitabine (1,250 mg/m^2^) based on their total body surface area (1,500 mg–2,400 mg) twice daily (after breakfast and within 30 minutes after dinner) for 14 days continuously followed by a 7-day off period. This 3-week period will comprise 1 course of treatment. Patients will receive 8 courses in total.

After completing all 8 courses, patients not exhibiting cancerous recurrence will undergo continuous monitoring without additional treatment.

**5-3-2. Group B: Study treatment group (16 courses [approx. 12 months])**

Capecitabine (Xeloda tablets): Patients will receive the established dosage of capecitabine (1,250 mg/m^2^) based on their total body surface area (1,500 mg–2,400 mg) twice daily (after breakfast and within 30 minutes after dinner) for 14 days continuously followed by a 7-day off period. This 3-week period will comprise 1 course of treatment. Patients will receive 16 courses in total.

After completing all 16 courses, patients not exhibiting cancerous recurrence will undergo continuous monitoring without additional treatment.

**5-3-3. Established capecitabine dosages**

The actual dosages of capecitabine administered are as follows.

No dose corrections due to changes in body weight are performed after protocol treatment initiation.

Table 2 Per-administration capecitabine dosages by subject total body surface area (Groups A and B)

| Body Surface Area | Single-dose Volume |
| --- | --- |
| ≤1.33 m^2^ | 1,500 mg (5 tablets) |
| ≥1.33 m^2^ ≤1.57 m^2^ | 1,800 mg (6 tablets) |
| ≥1.57 m^2^ ≤1.81 m^2^ | 2,100 mg (7 tablets) |
| ≥1.81 m^2^ | 2,400 mg (8 tablets) |

## **5-4. Treatment Adjustment Provisions**

**5-4-1. Treatment suitability criteria**

1) At the start of each course of treatment, administration is initiated after confirming that the examination values and clinical findings on the day of or day preceding the scheduled administration satisfy all suitability criteria. In addition, as appropriate during each course, administration can be continued after confirming that all suitability criteria are satisfied. (However, if clinical test results are not available on the subject’s visitation day, the attending physician may decide whether or not to administer the protocol treatment based on the most recent examination values.)

[Treatment suitability criteria]

WBC count ≥ 3,000/mm^3^

Eosinophil count ≥ 1,500/mm^3^

Thrombocyte count ≥ 75,000/mm^3^

Total serum bilirubin ≤ 1.5x hospital standard upper limit

AST, ALT ≤ 2.5x hospital standard upper limit

Serum creatinine ≤ 1.5x hospital standard upper limit

2) Grade I or lower non-hematological toxicity excluding alopecia or dysgeusia

**5-4-2. Off-drug periods/dose reductions/therapy reinitiation provisions**

Adverse events are evaluated based on the Japanese-language Common Terminology Criteria for Adverse Events (CTCAE) v3.0 Japan Clinical Oncology Group/Japan Society of Clinical Oncology (JCOG/JSCO) version. Off-drug periods, dose reduction, and therapy reinitiation due to the occurrence of adverse events during capecitabine administration are managed according to “Table 3 Off-period/dose reduction criteria” and “Table 4 Reduced dosages”. In addition, handling of Grade II/III hand-foot syndrome, diarrhea, nausea, and vomiting shall be performed in accordance with “5-4-3. Provisions for off-drug periods/dose reductions/therapy reinitiation following adverse events not falling under the above”.

Clinical laboratory values may also be treated as adverse events in the following cases only:

(1) In case of a serious adverse event

(2) In cases where dose reduction, an off-period, or discontinuation of the protocol treatment is necessary

(3) When medical treatment is necessary

(4) When determined to be clinically problematic

1) Warnings concerning off-drug periods, dose reductions, and therapy reinitiation provision are as follows.

(1) Off-drug periods and dose reduction owing to worsening complications observed before the start of administration may be arranged based on fluctuations in adverse event grade, if deemed appropriate by the attending physician. For example, if Grade I alkaline phosphatase before administration deteriorates to Grade II during capecitabine administration, this may be treated as a Grade I adverse event, understood as a shift in adverse event grade.

(2) If the attending physician determines that adverse events do not cause interruption or delays in administration, such as in cases of alopecia or dysgeusia, and it is determined that there is no possibility of the event becoming serious, administration of capecitabine may be continued at the same dosage without an off-period or dose reduction.

(3) When anemia is controllable by transfusion, there is no need for an off-period or dose reduction, and treatment may continue at the same dose.

(4) If adverse events of Grade II appear at the end of 2 weeks of capecitabine administration and recover to Grade 0–I during the 1 week off-period prescribed by the treatment protocol, “Table 3 Off-period/dose reduction criteria” does not apply, and treatment may continue without changes as deemed appropriate by the attending physician.

(5) An off-drug period should be considered during the 2 weeks of administration as a non-administration day and the administration schedule should be maintained (2 weeks on, 1 week off). Capecitabine not administered because of an off-drug period should not be re-administered. If the start of an individual treatment course is delayed, the 3-week following the start date shall constitute 1 course.

(6) If a dose reduction is performed, the dose should not be increased later.

(7) If treatment initiation is delayed for more than 3 weeks or an adverse event requiring discontinuation appears, the protocol treatment is deemed to have been discontinued.

(8) Although patient withdrawal based on self-determination is acceptable, such patients should be instructed to contact their attending physician or his/her proxy to the extent possible. The patient’s drug adherence should also be checked upon visitation.

2) The following are provisions for drug off-periods due to the occurrence of adverse events during treatment.

(1) When an adverse event of Grade I has appeared

Administration may be continued without changes.

(2) When an adverse event of Grade II or higher appears

Off-drug periods, dose reductions, and therapy reinitiation should be conducted according to “Table 3 Off-period/dose reduction criteria” below. For individual subjects, confirm the dose reduction based on the “registration confirmation form”. A single dose at the time of dose reduction and the number of single dose tablets are calculated based on “Table 4 Reduced dosages”. A single dose at the time of dose reduction is determined according to the subject’s total body surface area at the time of enrollment.

Table 3 Off-period/dose reduction criteria

|  |  | Countermeasures during treatment period | Treatment reinitiation dosages |
| --- | --- | --- | --- |
| Grade 1 |  | Off-period/dose reduction not needed | Dose reduction not needed |
| Grade 2 | 1st onset  2nd onset  3rd onset  4th onset | Off-drug until improvement to Grade 0-1  Off-drug until improvement to Grade 0-1  Off-drug until improvement to Grade 0-1  Suspend protocol treatment | Dose reduction not needed  Dose reduction 1  Dose reduction 2  － |
| Grade 3 | 1st onset  2nd onset  3rd onset | Off-drug until improvement to Grade 0-1  Off-drug until improvement to Grade 0-1  Suspend protocol treatment | Dose reduction 1  Dose reduction 2  － |
| Grade 4 |  | Suspend protocol treatment | － |

Note) Number of onsets refers to the number of appearances of events of the same grade, and is not limited to the number of appearances of the same event.

Table 4 Reduced dosages

| Body surface area | Single dose | | |
| --- | --- | --- | --- |
|  | Initial dose | Dose reduction 1 | Dose reduction 2 |
| ≤1.13 m^2^ | 1,500 mg (5 tablets) | 900 mg (3 tablets) | 600 mg (2 tablets) |
| ≥1.13 m^2^　≤1.21 m^2^ |  | 1,200 mg (4 tablets) |  |
| ≥1.21 m^2^　≤1.33 m^2^ |  |  | 900 mg (3 tablets) |
| ≥1.33 m^2^　≤1.45 m^2^ | 1,800 mg (6 tablets) |  |  |
| ≥1.45 m^2^　≤1.57 m^2^ |  | 1,500 mg (5 tablets) |  |
| ≥1.57 m^2^　≤1.69 m^2^ | 2,100 mg (7 tablets) |  |  |
| ≥1.69 m^2^　≤1.77 m^2^ |  |  | 1,200 mg (4 tablets) |
| ≥1.77 m^2^　≤1.81 m^2^ |  | 1,800 mg (6 tablets) |  |
| ≥1.81 m^2^ | 2,400 mg (8 tablets) |  |  |

**5-4-3. Provisions for off-drug periods/dose reductions/therapy reinitiation following adverse events not falling under the above**

1) Grade II/III hand and foot syndrome

Adverse event grade is determined based on the criteria in “Table 5 Diagnostic criteria for hand-foot syndrome” below and subject to “Table 3 Off-period/dose reduction criteria” above.

Table 5 Diagnostic criteria for hand-foot syndrome

| Grade | Clinical area | Functional area |
| --- | --- | --- |
| 1 | Numbness, skin hypersensitivity, burning, tingling, painless swelling, painless erythema | Everyday activities are not impaired |
| 2 | Swelling accompanying painful skin erythema | Everyday activities are somewhat impaired |
| 3 | Moist desquamation, ulcers, blisters, strong pain | Everyday activities are impossible |

If the grade of the corresponding symptom is inconsistent between the diagnostic areas (clinical area, functional area), adopt a grade that can be determined to be more suitable.

This criterion applies only to the diagnosis of hand-foot syndrome, and not to diagnoses of skin or other organ-related symptoms.

2) Grade II/III diarrhea, nausea, and emesis

Discontinue capecitabine use and take appropriate countermeasures.

If symptoms can be controlled with treatment intervention within 2 days, the protocol treatment may be resumed without changes. If control requires more than 3 days, “Table 3 Off-period/dose reduction criteria” should be followed (including diarrhea determined to require more than 3 days for diagnosis or treatment due to the possibility of dihydropyrimidine dehydrogenase [DPD] deficiency, etc.)

## **5-5. Treatment Discontinuation Provisions**

**5-5-1. Treatment discontinuation criteria**

If applicable, the attending physician will discontinue subsequent treatments and take appropriate measures. Investigators should observe and inspect for the evaluation items specified in “Table 6 Examination items and evaluation schedule” below at the time of discontinuation and record their findings and the accompanying rationale in their case report. In cases of termination, the attending physician shall promptly submit an administration progress survey to the study secretariat via fax.

1) When a subject him/herself withdraws consent

2) When a subject him/herself requests discontinuation of the protocol treatment

3) When recurrence of the original disease is observed

4) When novel cancerous lesions (duplicated cancer or multifocal colorectal cancer [excluding M cancer]) occurs

5) When the attending physician determines that protocol continuation will be difficult due to deterioration of comorbidities or onset of complications

6) When there is no relief or disappearance of symptoms following withdrawal due to an adverse event that falls under the off-period/dose reduction provisions (delay of treatment start by over 3 weeks or withdrawal)

7) If administration cannot be continued due to the occurrence of an adverse event even after 1 or 2 level reductions under the off-period/dose reduction provisions

8) If an adverse event of Grade IV occurs, the protocol treatment must be discontinued. (If capecitabine treatment is restarted at the discretion of the attending physician after protocol termination, a case report must be submitted.)

9) When the attending physician determines that protocol continuation will be difficult owing to an adverse event

10) If a subject dies

11) When the protocol treatment cannot be continued because the subject was transferred to a hospital

12) If a subject is determined to be ineligible after enrollment

13) Other cases in which the attending physician deems it necessary to terminate the protocol treatment

**5-5-2. Procedures when the protocol treatment is suspended**

In cases in which the treatment protocol is suspended or cases of the presence of novel cancerous lesions or recurrence 5 years after the last subject enrollment registration is detected, outcome observation is continued, with the exception of cases meeting the following criteria:

1) When a subject withdraws consent to follow-up after withdrawal from the study

2) If a subject dies

3) When tracking a subject is difficult for other reasons

## **5-6. Safety Concerns**

Capecitabine is expected to be administered on an outpatient basis, and safety issues can rapidly become severe if adverse events such as diarrhea occur. If the attending physician observes an adverse event before a subject’s scheduled visit, the attending physician should immediately contact the hospital and instruct the hospital to provide treatment.

Many adverse events associated with capecitabine are reversible and the treatment protocol need not be discontinued, but an off-drug period or dose reduction may be required.

**1) Diarrhea**

Capecitabine has been reported to occasionally cause severe diarrhea. If severe diarrhea occurs, symptomatic treatments such as electrolyte rebalance by infusion may be necessary. If nonsevere diarrhea occurs, administration of common anti-diarrheal agents such as loperamide is effective.

**2) Nephropathy**

The incidence of Grade III/IV adverse events has been found to be higher in subjects with moderate renal dysfunction (creatinine clearance: 30–50 mL/min) compared to those with normal renal function. In this study, subjects with moderate to severe renal dysfunction (creatinine clearance ≤ 50 mL/min) before treatment initiation will be disqualified.

Dose adjustment is unnecessary in subjects with mild renal impairment (creatinine clearance 51–80 mL/min).

**3) Cardiotoxicity**

Myocardial infarction, myocardial ischemia, angina pectoris, arrhythmia, cardiac arrest, heart failure, sudden death, electrocardiogram abnormality, and cardiomyopathy have been observed in patients receiving capecitabine only. These findings are similar to cardiotoxic events reported overseas to be associated with fluoropyrimidine drugs. Care must be taken when administering capecitabine to patients with a history of cardiovascular disease.

**4) Motor Impairment Syndrome**

The severity of hand-foot syndrome is classified from Grade I-III (Table 5). In cases of Grade II/III hand-foot syndrome, “Table 3 Off-period/dose reduction criteria” should be followed.

## **5-7. Concomitant Treatments**

**5-7-1. Contraindicated drugs and therapies**

Do not concomitantly administer other anticancer drugs, immunotherapies, endocrine therapies, unapproved drugs, drugs considered to affect the evaluation of radiation therapies, or experimental drugs.

**5-7-2. Drugs subject to concomitant usage warnings**

1) Oral coumarin anticoagulants

In patients receiving capecitabine and oral coumarin anticoagulants concomitantly, observe coagulation parameters (prothrombin time) frequently to appropriately adjust the anticoagulant dosage. In clinical pharmacology studies, clinically important drug interactions have been observed with respect to concomitantly administered capecitabine and warfarin. Patients taking capecitabine in combination with coumarin anticoagulants such as warfarin and phenprocoumon were reported to exhibit fluctuations in coagulation parameters and bleeding, and cases of patient death have also been reported. Examples have been reported in postmarketing data of cases in which clinically significant prothrombin time prolongation and international normalized ratio (INR) elevation occurred when patients who were stable using anticoagulant began taking capecitabine. These symptoms manifest several months after the initiation of capecitabine administration, and in several cases they also occur within 1 month after discontinuation of administration. Furthermore, these events are not affected by liver metastasis. Patient age of over 60 years and onset of cancer are both risk factors for the development of coagulopathy. Use of low molecular weight heparin instead of coumarin is safe under the guidance of a physician.

2) Phenytoin

Increase in plasma phenytoin concentration has been reported as a result of concomitant use of the antiepileptic drug phenytoin and oral capecitabine. Accordingly, when capecitabine and phenytoin are used in combination, plasma phenytoin concentration should be observed periodically, and patients should be monitored for subjective symptoms, and changes in clinical laboratory values.

**5-7-3. Safe drugs for concomitant use and recommended supporting treatments**

Drugs other than those specified in “Contraindicated drugs and therapies” may be used concomitantly based on the judgment of the attending physician. For concomitant medications and concomitant treatments (including treatment, etc.), describe the name of the medicine (treatment), the period of the combination use, and the reason for the combination in the case report.

1) Supportive care for hand-foot syndrome

There is currently no established treatment method, but as symptomatic treatment, topical steroid preparations or moisturizing creams are commonly applied. Improvement of symptoms of hand-foot syndrome has been reported when vitamin B6 (50–150 mg, twice daily) is taken during continuous infusion of 5-FU 23). When vitamin B6 is administered, the contents of the treatment should be specified in the case report.

As for the presence or absence of the prophylactic administration of vitamin B6 formulation for hand-foot syndrome, both positive and negative reports have been reported ^24-30)^. When used, utilize with application of moisturizing cream prior to the appearance of symptoms.

- Vitamin B6 formulation (60 mg/day) administered daily (continued even during capecitabine withdrawal)
- Frequent application of moisturizing cream (estimate: 5 times or more per day)

[Reference: National Health Insurance approved dose of vitamin B6 preparation]

Pyridoxal phosphate (Pydoxal tablets): 60 mg/2 times/day

Pyridoxine hydrochloride (Aderoxin tablets): 100 mg/day

Pyridoxal hydrochloride calcium powder (Aderoxal powder): 60 mg/3 times/day

2) Supportive care for diarrhea

For diarrhea, loperamide, etc. may be given, and preventive measures after onset can also be taken. In serious cases, appropriate measures such as infusion should be taken.

3) Supportive care for nausea and vomiting

For nausea/vomiting, 5-HT3 formulations such as granisetron hydrochloride and other antiemetic drugs may be used, and preventive measures after onset can also be taken.

4) Combination therapy for neutropenia

In principle, the dosing criteria for G-CSF preparations (such as renograstim) should be as described in the package insert, and prophylactic administration should not be performed. G-CSF preparations may be administered at the discretion of the attending physician in cases in which a decrease in neutrophil count or WBC count of Grade III or higher with fever or a decrease in neutrophil count or WBC count of Grade IV is observed.

5) Combination therapy for fever/infection

If infection accompanied by fever is suspected, antibiotics should be administered. In principle, prophylactic administration of antibiotics is not performed.

## **5-8. Post-treatment**

Post-treatment should not be performed to address underlying illnesses (chemotherapy, endocrine therapy, molecularly targeted therapy, immunotherapy, etc.) until cancerous recurrence is confirmed after termination of the protocol treatment. Post-treatments administered after completion or termination of the protocol treatment and after confirmation of recurrence are not specified.

# **6. Examination/Observation Items**

## **6-1. Pre-Enrollment Examination/Observation Items**

1) Patient background

Sex, age at enrollment, date of written consent acquisition, past medical history, etc.

2) Lesions and evaluation

Histopathological findings of primary lesion (occupied area, tissue type, depth of wall invasion, number of lymph node searches, lymph node metastasis (number, N degree: N1, N2, N3), lymph vascular invasion, presence or absence of venous invasion, date of surgery, presence or absence of preoperative complications, presence or absence of postoperative complications, surgical operation type, observation method (laparotomy, laparoscope), degree of lymph node dissection, etc.

3) Pregnancy test (if applicable)

4) Treatments for comorbidities, complications

5) Electrocardiogram (ECG) * Preoperative examination also counts

6) Chest simple radiographic examination, abdominal ultrasonography or abdominal computed tomography (CT) * Examination can be performed before surgery

7) Vital signs and performance status (PS)

Height, weight, PS

8) Hematological examination (within 14 days before enrollment)

RBC count, hemoglobin, hematocrit, WBC count, leukocyte fraction, platelet count

9) Blood biochemistry testing (within 14 days before enrollment)

Serum total bilirubin, AST, ALT, ALP, total protein, serum creatinine

10) Tumor markers (within 14 days before enrollment)

CEA, CA 19-9

11) Subjective symptoms

## **6-2. Examination/Observation Items During Treatment Protocol and Observation Periods**

During this clinical trial, the following tests and observations are periodically performed and evaluated according to the safety evaluation schedule and the relapse evaluation schedule. For details of the timing of each inspection and observation, refer to “Examination items and evaluation schedule”. For hematology/blood biochemistry testing and adverse events, report the worst grade observed during each course in the “Treatment Course Report Form” (adverse event: clinical laboratory test value, subjective findings)”.

1) PS

2) Hematology tests

RBC count, hemoglobin, hematocrit, WBC count, WBC fraction, thrombocyte count

3) Blood biochemistry tests

Total serum bilirubin, AST, ALT, ALP, total plasma protein, serum creatinine

4) Tumor markers

CEA, CA19-9

5) Simple chest radiography or chest CT

6) Abdominal ultrasound, abdominal CT

7) Colonoscopy

If preoperative colonoscopy findings are inadequate, a contrast colon examination or colonoscopy is performed within 6 months postoperatively and the remaining colon is examined.

8) Adverse events

Registered cases are determined based on the Common Terminology Criteria for Adverse Events (CTCAE) V 3.0 adverse event criteria (Appendix 2 for filling out case record forms).

“Adverse event” refers to any undesirable or unintended signs (including anomalies in clinical laboratory values), symptoms or diseases that occur when a pharmaceutical is administered, and a causal relationship with the study drug; the presence or absence of the study drug does not matter.

Self-cognitive symptoms should be observed during the administration period and up to 28 days after the last administration and recorded according to the adverse event criteria of the CTCAE V 3.0. Based on the judgment of the attending physician, adverse events not described by this standard are evaluated on a grade scale: I: mild, II: moderate, III: more severe, IV: severe.

9) Drug adherence

Dose initiation date, daily dosage, medication days, missed or reduced doses for each course and reasons

Notwithstanding the above, other clinical examinations deemed clinically necessary are also conducted as appropriate.

## **6-3. Checking Procedures for Recurrent and Non-Recurrent Cancerous Lesions**

As a general rule, subjects are examined every 3 months until postoperative day 3, and every 6 months from post-study year 3 onward for cancerous recurrence and appearance of tumor markers. In addition, imaging diagnoses are performed in the form of chest radiography or CT, and abdominal ultrasound examination or CT is performed every 6 months until postoperative year 5. When recurrence is suspected, recurrence is confirmed using appropriate imaging diagnoses such as colonoscopy and CT.

Imaging diagnostics for confirming recurrence include the following:

(1) Liver metastasis: ultrasonography or CT, magnetic resonance imaging (MRI)

(2) Lung metastasis: chest radiographic examination or CT

(3) Abdominal lymph node metastasis: ultrasonography or CT

(4) Local recurrence: CT, MRI, intestinal radiography, or endoscopy

The date of confirmation of recurrence and the confirmation method should also be included in the report submitted.

Document the presence/absence of nonrecurrent cancerous lesions, the site of detection (if applicable), and the date of confirmation in the report.

*M cancer is not treated as a cancerous lesion.

## **6-4. Outcome Investigation**

Outcome investigation (confirmation of survival) of enrolled subjects is conducted annually (every year). Remote outcomes such as survival may also be investigated following the end of the study period.

## **6-5. Discontinuation of Treatment Protocol**

1) Last day of drug administration

2) Date of decision to discontinue treatment

3) Reason for discontinuing treatment protocol

Table 6 Examination items and evaluation schedule

|  | Pre-enrollment | Postoperative Course (months) | | | | | | | | | | | |
| --- | --- | --- | --- | --- | --- | --- | --- | --- | --- | --- | --- | --- | --- |
|  |  |  | 1 year | | 2 years | | 3 years | | 4 years | |  | 5 years | |
|  |  | 3 6 9 12 | | 3 6 9 12 | | 3 6 9 12 | | 3 6 9 12 | | 3 6 9 12 | | | Months |
| Consent acquisition | ● |  | |  | |  | |  | |  | | |  |
| Patient background | ● |  | |  | |  | |  | |  | | |  |
| Disease assessment | ● |  | |  | |  | |  | |  | | |  |
| Pregnancy check (if necessary) | ● |  | |  | |  | |  | |  | | |  |
| Concomitant illnesses, treatments | ● |  | |  | |  | |  | |  | | |  |
| ECG | ● |  | |  | |  | |  | |  | | |  |
| Vital signs, PS | ● | In following with Table 7 | |  | |  | |  | |  | | |  |
| Hematological testing | ● |  |  |  | |  | |  | |  | | |  |
| Blood biochemistry testing | ● |  |  |  | |  | |  | |  | | |  |
| Adverse events and countermeasures |  |  |  |  | |  | |  | |  | | |  |
| Drug adherence |  |  |  |  | |  | |  | |  | | |  |
| Interviews/examinations |  | ● ● ● ● | | ● ● ● ● | | ● ● ● ● | | ● ● | | ● ● | | |  |
| CEA, CA 19-9 | ● | ● ● ● ● | | ● ● ● ● | | ● ● ● ● | | ● ● | | ● ● | | |  |
| Chest X-ray  CT | ● | ● ●  or or  ● ● | | ● ●  or  ● | | ● ●  or  ● | | ● ●  or  ● | | ● ●  or  ● | | |  |
| Abdom. ultrasound  CT | ●  or  ● | ● ●  or or  ● ● | | ●    ● | | ●    ● | | ●    ● | | ●    ● | | |  |
| Pelvic CT (colorectal cancer) |  | ●* ● | | ●* ● | | ● | | ● | | ● | | |  |
| Colonoscopy |  | ● | | ● | | ● | |  | |  | | |  |
| MRI* |  |  | |  | |  | |  | |  | | |  |

*: As necessary #: Search for cancerous recurrence at anastomosis site

Table 7-1 Group A: Standard-of-care group

|  | Postoperative Course (months) | | | | | | | | | | | | | | | | | |
| --- | --- | --- | --- | --- | --- | --- | --- | --- | --- | --- | --- | --- | --- | --- | --- | --- | --- | --- |
|  | 1 | |  | 3 | | |  | 6 | | |  | | 9 |  | | | | 12 |
| Dosing cycle | 1 | | 2 | 3 | 4 | 5 | 6 | 7 | 8 | - | - | - | - | - | - | - | - | |
| Weeks since treatment initiation | 1 | 2 | 4 | 7 | 10 | 13 | 16 | 19 | 22 | - | - | - | - | - | - | - | - | |
| PS |  | △ | ● | ● | ● | ● | ● | ● | ● |  | | | | | | | | |
| Hematological tests |  | △ | ● | ● | ● | ● | ● | ● | ● |  | | | | | | | | |
| Blood biochemistry tests |  | △ | ● | ● | ● | ● | ● | ● | ● |  | | | | | | | | |
| Adverse events and countermeasures | Subjects were monitored and data was recorded during the treatment period and for 28 days after completion of treatment | | | | | | | | |  | | | | | | | | |
| Drug adherence |  | | | | | | | | |  | | | | | | | | |

Table 7-2 Group B: Study treatment group

|  | Postoperative Course (months) | | | | | | | | | | | | | | | | | | |
| --- | --- | --- | --- | --- | --- | --- | --- | --- | --- | --- | --- | --- | --- | --- | --- | --- | --- | --- | --- |
|  | 1 | |  | 3 | | |  | 6 | | | | |  | 9 | | |  | | 12 |
| Dosing cycle | 1 | | 2 | 3 | 4 | 5 | 6 | | 7 | 8 | 9 | 10 | | 11 | 12 | 13 | 14 | 15 | 16 |
| Weeks since treatment initiation | 1 | 2 | 4 | 7 | 10 | 13 | 16 | | 19 | 22 | 25 | 28 | | 31 | 34 | 37 | 40 | 43 | 46 |
| PS |  | △ | ● | ● | ● | ● | ● | | ● | ● | ● | ● | | ● | ● | ● | ● | ● | ● |
| Hematological tests |  | △ | ● | ● | ● | ● | ● | | ● | ● | ● | ● | | ● | ● | ● | ● | ● | ● |
| Blood biochemistry tests |  | △ | ● | ● | ● | ● | ● | | ● | ● | ● | ● | | ● | ● | ● | ● | ● | ● |
| Adverse events and countermeasures | Subjects were monitored and data was recorded during the treatment period and for 28 days after completion of treatment | | | | | | | | | | | | | | | | | | |
| Drug adherence |  | | | | | | | | | | | | | | | | | | |

●: Necessary item　　△: Performed as necessary

# **7. Study Endpoints**

## **7-1. Primary Endpoint**

**7-1-1. Disease-free survival (DFS)**

1) Definition

The period from the enrollment date to the date of the first event confirmation.

2) Event definition

An event is defined as an instance of any of the following:

(1) Recurrence

(2) First occurrence of non-recurrent cancer lesions after the date of enrollment

(M cancer is not treated as a cancerous lesion)

(3) Death from any cause

Surviving subjects not deemed to exhibit cancerous lesions other than recurrences will be determined to have no cancerous lesions as of the final monitoring date.

3) Definition of relapse and recurrence date

Recurrence findings that fall under any of the following are defined as "recurrence". However, if possible, it is advisable to check recurrence via biopsy.

(1) Imaging diagnosis: Confirmation of recurrence based on imaging findings; the date diagnosis was determined is considered the recurrence date.

(2) Pathological diagnosis: If diagnosis of recurrence is made clinically prior to biopsy, the date of diagnosis is considered the recurrence date. If diagnosis could not be made and biopsy enabled diagnosis of recurrence, the biopsy date is considered the recurrence date.

(3) Clinical judgment: If recurrence cannot be confirmed by imaging diagnosis or pathological diagnosis because of rapid deterioration or transference, and clinical findings are reference only, the date of confirmation shall be the recurrence date. However, in such cases, the clinical findings that resulted in the judgment of recurrence should be recorded in the case report along with the confirmation date.

Recurrence does not include increases in tumor marker values.

## **7-2. Secondary Endpoints**

**7-2-1. Relapse-free survival (RFS)**

1) Definition

The period from the enrollment date to the date of the first event confirmation.

2) Event definition

An event is defined as an instance of any of the following:

(1) Recurrence

(2) Death from any cause

Surviving subjects not deemed to exhibit cancerous lesions other than recurrences will be determined to have no cancerous lesions as of the final monitoring date.

**7-2-2. Overall survival (OS)**

1) Definition

The period from the enrollment date to the date of patient death from any cause.

2) Event Definition

An applicable event is defined as an instance of any of the following:

Patient death from any cause; in the case of surviving subjects, the last confirmed date of survival, and in the case of the non-traceable subjects, the last confirmed day of survival before becoming non-traceable.

**7-2-3. Adverse event onset rate and severity (safety evaluation)**

The incidence of adverse events was evaluated. Evaluation of adverse events is performed according to Japan Clinical Oncology Group/Japanese Society of Clinical Oncology (JCOG/JSCO) version of the Common Terminology Criteria for Adverse Events (CTCAE) v3.0.

**7-2-4. Drug adherence assessment**

Oral compliance during treatment is evaluated by the ratio of the amount of drug per unit time (Individual Dose Intensity: IDI) per unit time to the actual dose per actual administration period. However, IDI is defined as follows using the prescribed administration period and the scheduled dose:

- IDI = (actual total dose/actual administration period)/(expected total dose/scheduled administration period)

The scheduled administration period and the scheduled dose are defined as shown in the table below for Group A and Group B, respectively.

|  | Group A | Group B |
| --- | --- | --- |
| Planned dosage | 1 dose of capecitabine based on body surface area×2×planned treatment period (1 course)×14 | |
| Planned dosing period | 24-week period  (8 courses) | 48-week period  (16 courses) |

**7-2-5. HRQOL (Health-Related Quality of Life)/medical economics *tertiary studies conducted by participating study sites**

HRQOL and medical economics surveys were conducted at participating facilities. Models were evaluated based on collected survey data from the perspective of medical economics. Details are specified separately.

# **8. Statistical Matters (Research Evaluation Criteria and Analyses)**

## **8-1. Definition of the Target Population for Analysis**

1) All enrolled subjects: The group of all enrolled subjects excluding duplicate or invalid enrollments.

2) All eligible subjects: The group of all enrolled subjects excluding “ineligible subjects”. However, ineligible subjects are defined as subjects determined to not meet the selection criteria after enrollment, or cases in which it becomes clear that the subject meets 1 or more of the exclusion criteria.

3) All treated subjects: The group of all enrolled, qualified subjects excluding those for whom the protocol treatment was not completed.

Based on the above definitions, the main subject groups targeted for the analysis of efficacy and safety shall be all enrolled subjects and all treated subjects.

## **8-2. Primary Endpoint Analyses**

1) The main objective of this study is to evaluate the superiority of the study drug in terms of the disease-free survival (DFS) of group B (test treatment group: 16 courses administered) compared with group A (standard treatment group: 8 courses administered). The median annual DFS for all enrolled subjects is estimated by group using the Kaplan-Meier method. Greenwood’s formula is used for interval estimation. For the evaluation of the hypothesis, the stratified log-rank test, which considers a layer composed of a combination of allocation adjustment factors excluding study sites, is used. If the one-sided significance level is 5% and it is demonstrated that group B exhibits statistically significantly superior performance compared with group A, group B will be regarded as the standard therapy replacing the group A therapy. If group B’s performance is inferior to group A, the standard therapy will remain unchanged.

2) The stratified Cox proportional hazard model will be used. This model considers the layer composed of the combination of allocation adjustment factors other than study site as an estimate of the treatment effect. The hazard ratio of the treatment effect among the groups and its 95% confidence interval will also be obtained. A Wald-type estimator is used to estimate confidence intervals. If necessary, in addition to the allocation adjustment factor, an adjusted hazard ratio accounting for background bias will be derived.

3) A similar secondary analysis will be carried out for all eligible subjects.

## **8-3. Secondary Endpoint Analyses**

1) DFS, relapse-free survival (RFS), and overall survival (OS) are evaluated using the same analysis.

2) An estimate of 2-year DFS rate is derived from the DFS Kaplan-Meier curve.

3) Adverse events are tabulated for each group, and the onset frequency of adverse events in the standard treatment group and the test group will be compared. However, no hypothesis testing will be conducted.

4) Drug adherence is calculated based on the rate of individual drug adherence and actual dosage per actual administration period, and is compared between the groups.

5) Summary statistics regarding total treatment costs will be estimated and compared between the groups. However, no hypothesis testing will be conducted.

## **8-4. Target Number of Subjects and Statistical Basis**

Number of target subjects: 1,200 subjects (1 group: 600 subjects)

The main objective of this study is to evaluate the superiority of the study drug in terms of the DFS of group B (test treatment group: 16 courses administered) compared with group A (standard treatment group: 8 courses administered). There is no difference in disease-free survival (DFS) during this 6-month period since group A and group B receive essentially the same 6-month treatment after randomization, and differences in DFS between the groups are expected to appear 6 months after randomization.

When designing the number of subjects assuming a normal exponential distribution without considering the above, the number of required subjects is underpowered. Therefore, in this study, the number of subjects was established assuming a segmented exponential model. In the 6 months following randomization, the hazard ratios for DFS in group A and group B were the same, and assuming that differences in DFS begin to appear after 6 months, the 5-year DFS rate in group A will be 56–62%. In group B, if 5% or 7% is expected to be added to the 5-year DFS rate of group A, the number of required subjects is as follows, with a one-sided significance level of 5% and a detection power of 80% (Table 8-1). The power function of the Statistical Analysis System (SAS) ver.9.1 was used to determine the appropriate number of subjects for this study.

Table 8-1 With a 2-year enrollment period and 5-year follow-up period

| 5-year disease-free survival rate (Group A) | 5-year disease-free survival rate (Group B) | |
| --- | --- | --- |
|  | 5% increase | 7% increase |
| 56% | 2,394 patients | 1,206 patients |
| 58% | 2,336 patients | 1,176 patients |
| 60% | 2,272 patients | 1,142 patients |
| 62% | 2,202 patients | 1,104 patients |

Table 8-2 With a 3-year enrollment period and 5-year follow-up period

| 5-year disease-free survival rate (Group A) | 5-year disease-free survival rate (Group B) | |
| --- | --- | --- |
|  | 5% increase | 7% increase |
| 56% | 2,288 patients | 1,152 patients |
| 58% | 2,228 patients | 1,120 patients |
| 60% | 2,164 patients | 1,086 patients |
| 62% | 2,092 patients | 1,048 patients |

Since the 5-year DFS rate in the X-ACT study was 60.8%, assuming that the 5-year survival rate for group A is 60% and that the 5-year survival rate in group B is 67%, with 2 years of enrollment and 5 years of follow-up, a detection power 80%, and a one-sided significance level of 5%, 1,142 subjects (480 events) across the 2 groups are necessary. However, the number of required events was estimated based on a simulation. Also, contrary to expectations, even if the 5-year survival rate for group A is 56%, a detection power of 74% can be achieved with 480 events.

Assuming a dropout rate during the test period of approximately 5%, the number of target subjects in the 2 study groups is set to 1,200 (600 per group).

## **8-5. Revision to the Number of Subjects**

Patient prognosis in Japan may be better than that of the X-ACT study owing to differences in patient background and surgical results. Therefore, we estimated DFS Kaplan-Meier curves for both groups using the latest data as early as possible before enrolling the target number of subjects and compared the curved against those of the X-ACT. As a result of comparing the annual survival rate, patient background, etc. with the results of the X-ACT trial, the number of subjects in this study will be revised if it is determined that event occurrences are clearly inadequate.

When redesigning the number of subjects, the magnitude that appears to be clinically appropriate will be reviewed as an additional effect expected for group B against the 5-year DFS rate for group A.

## **8-6. Study Period**

Enrollment period: 2 years (Sept. 2008–Aug. 2010)

Follow-up period: 5 years after last enrollment

Cumulative study period: 7 years (Sept. 2008–Aug. 2015)

The registration and follow-up periods may be extended if the number of enrolled subjects is less than the target number or if the number of events is less than the required number.

## **8-7. Interim Analyses and Early Study Termination**

Three interim analyses were conducted for the purpose of evaluating whether enrollment can be continued and whether the primary endpoint of the study has been achieved. The first interim analysis will be performed after half of the number of subjects are enrolled. This analysis will comprehensively evaluate whether enrollment should be continued. However, the effectiveness of the first intermediate analysis is not evaluated. As a general rule, registration will not be suspended during interim analysis.

In the second interim analysis, the main purpose is to evaluate study effectiveness, and this analysis is performed after the 1,200 subjects are enrolled. The second interim analysis will also involve an exploratory analysis that will serve as a reference for the secondary comparative study of the 2-year DFS rate. In this exploratory evaluation, the selection design ^31)^ described by R. Simon is applied, and the selection results are disclosed only to the members of the Data and Safety Monitoring Committee and the researchers recognized by the Executive Committee. However, this disclosure will not affect the verifiability of the primary endpoint of this study, and these results will also be published in the interim analysis.

In the third interim analysis, the main purpose is to evaluate the effectiveness of the study, and this analysis will be conducted after half the number of necessary events (240 events) are observed during the follow-up period. If it is determined that the primary endpoint of the study has been achieved during interim analysis, the study will be terminated and the results of the study will promptly be made public at academic symposia and in academic journals.

Interim analyses are carried out by the data center. To maintain the overall Type I error rate at 5%, test multiplicity for the interim and final analyses was adjusted using Lan & DeMets’ α consumption function ^32)^. The difference in DFS between the groups was examined for statistical significance, but here the O’Brien & Fleming type was used as the α consumption function. During intermediate analysis, when the DFS for group B exceeded that of group A and the one-sided *p*-value of the stratified log-rank test fell below the significance level prescribed by the above method, the result was determined to be statistically significant and the test was terminated as a general rule. If the survival curve for group B is lower than that of group A, judgments are not made based on test results, and the issue of whether to comprehensively terminate the study is made with reference to estimated Bayes flow probability or conditional detection ability.

# **9. Emergency Reporting of Adverse Events**

If an adverse event falling under the following categories is observed, the supervising study investigator at the relevant study site shall report the event to the study secretariat. Such events are also reported to the highest supervising official at each study site. Various other reports are made to regulatory authorities pursuant to applicable ordinances and regulations, such as spontaneous reports from medical institutions to the Pharmaceutical Affairs Bureau at MHLW in accordance with the “Pharmaceuticals and Medical Devices Safety Information Reporting System” and voluntary reports from medical institutions to companies under the “Company Reporting System”.

## **9-1. Events Warranting Emergency Reporting**

**9-1-1. Any subject death occurring during the treatment protocol or within 30 days after the final day of drug administration**

Emergency reporting is required regardless of whether there is a causal relationship with protocol treatment. In cases of protocol treatment discontinuation, even if post-treatment has already been initiated, the event is subject to emergency reporting requirements if the event occurs within 30 days from the final day of protocol treatment administration. “Thirty days” refers to 30 calendar days counted from the final day of protocol treatment administration.

**9-1-2. Unknown Grade 4 non-hematological toxicity**

“Unknown adverse events” are adverse events not described in a product’s package insert or prescribing information.

**9-1-3. Life-threatening adverse events**

“Life-threatening adverse event” refers to known Grade IV hematological toxicities, known Grade 4 non-hematological toxicities, and fever/bleeding symptoms.

**9-1-4. Persistent or severe Health conditions**

Aplastic anemia, myelodysplastic syndrome, secondary cancers, etc.

## **9-2. Reporting Procedure**

In the event of an adverse event that is subject to emergency reporting requirements, the attending physician will promptly inform the study site supervisor. The study site supervisor will orally report to the study secretariat within 24 hours if adverse events subject to emergency reporting are observed. Furthermore, within 72 hours after adverse event discovery, the required items shall be recorded on an “Emergency Adverse Event Reporting Form” (APPENDIX 1: in-hospital reporting is also possible) and sent via fax to the study secretariat.

## **9-3. Management by the Study Secretariat**

**9-3-1. Determinations that termination of enrollment or communication with a study site is necessary**

The study secretariat, which receives reports from the study site supervisors, can request the opinion of these supervisors or their representatives regarding the urgency, importance, degree of impact, etc., of the report content, and can suspend or mandate countermeasures to be taken at a specific study site as necessary.

**9-3-2. Reports to the data and safety monitoring committee**

If an emergency adverse event reported by a study site is determined to be an adverse event requiring reporting to all participating study sites, the study secretariat will report such information to the Data and Safety Monitoring Committee within 15 days of discovery of such an adverse event. At the same time, the study secretariat will request that the study representative provide an opinion and review the adequacy of the response to adverse events.

**9-3-3. Reviews by the Data and Safety Monitoring Committee**

The Data and Safety Monitoring Committee reviews the content of adverse event reports and makes recommendations to the study representative regarding the future handling of such events, including whether to continue subject enrollment.

# **10. Data Collection and Retention**

## **10-1. Submission of Case Report Forms, Etc.**

The process for submission of Case Report Forms (CRFs) is as follows. Apart from the CRF, various survey forms are sent to and from the study secretariat in conjunction with subject enrollment, treatment, and subject monitoring/follow-up. (For the submission period and submission deadline, refer to the documentation regarding entering submitting CRFs, etc., attached.)

1) Subject Enrollment Form (distributed beforehand to participating facilities)

2) Case Report Form

Pre-treatment Report Form

Treatment Progress Report Form (drug/adverse event: clinical examination/adverse event: subjective finding)

Monitoring Form

3) Questionnaire Form

Administration Start Questionnaire

Treatment Course Dosage Questionnaire

Contact Form for Termination of Administration

Monitoring Period Questionnaire

4) Emergency Adverse Event Reporting Form

5) Standard Study Site Questionnaire

6) Notification of Change in Study Subject Physician

## **10-2. Recordkeeping and Retention Provisions**

CRFs, etc., are retained in the study secretariat office maintained by the Japanese Foundation for Multidisciplinary Treatment of Cancer (JFMC) for 10 years after completion of the study.

# **11. Ethical Matters**

## **11-1. Compliance Requirements**

This study will be conducted in compliance with the Declaration of Helsinki and the relevant ethical guidelines regarding clinical research (MHLW Notification No. 459, December 28, 2004).

## **11-2. Informed Consent**

**11-2-1. Consent acquisition period**

Subject consent to participation in this study will be obtained prior to enrollment.

**11-2-2. Consent Acquisition Procedure**

Prior to enrollment, the prospective subject him/herself will refer to written briefing materials that explain the purpose of the study, the study treatments, adverse events, the study period, the fact that study participation will not incur costs borne by the subject, and that refusal to participate or withdrawal of consent at a later date will be met with no disadvantages or retaliation. Next, the attending physician will explain the content of the consent form to the prospective subject and record the date of explanation and his/her signature as well as the signature of the subject. One copy of the consent form, as well as the briefing materials, is retained by the patient, and 1 copy is retained in accordance with the study protocol and the policies of the study site.

## **11-3. Retention of Subjects’ Personal Information and Subject Identifiability**

**11-3-1. Identifiability of subjects’ clinical data**

In submitting a CRF or the various other reports to study sites or the study secretariat, when identifying an enrolled subject, the subject’s case registration number or similar number issued at the time of enrollment should be used, and any information that could potentially be used by a third party to identify the subject should not be used. Investigators should also refrain from informing the study secretariat of the name of the subject(s) in question.

## **11-4. Protocol Adherence**

Researchers participating in this study will comply with this study protocol to the extent that such compliance does not unduly threaten the safety and human rights of patients.

## **11-5. Study Site Institutional Review Board (IRB) Approval**

In order to participate in this study, the institutional review board (IRB) of each study site must approve this study protocol and its accompanying patient briefing/consent documentation.

# **12. Study-related Expenses and Compensation**

## **12-1. Funding Sources and Financial Relationships**

This study will be conducted as a multicenter joint clinical trial organized by the Japanese Foundation for Multidisciplinary Treatment of Cancer (JFMC).

A research grant in an amount corresponding to the number of enrolled subjects will be paid to participating facilities by the JFMC.

## **12-2. Study-related Expenses**

This test is conducted within the scope of normal health insurance policies, and subjects’ health insurance will be applied to costs arising from the observations/examinations conducted during the study period.

## **12-3. Injury-related Compensation**

In the event of a health hazard arising from the implementation of this study, the attending physician and the participating study site will respond such that appropriate treatment and other necessary measures can be taken. However, health insurance will be applied to treatment provided, and monetary compensation will not be provided.

# **13. Amendments to the Clinical Study Protocol**

If it is necessary to amend this study protocol or patient briefing materials during assessment, the study representative will revise such materials with the approval of the Clinical Trial Review Committee. The amended study protocol or patient briefing materials shall be approved by the Ethics Committee (or IRB) of each study site.

This does not apply to protocols that do not carry the possibility of increasing the risk borne by patients participating in the study and that are not related to the primary endpoint of the study.

If it is necessary to amend this study protocol, participant enrollment shall be temporarily suspended and resumed after revisions are completed. However, this policy does not apply if the proposed amendment does not directly affect subject enrollment.

# **14. Monitoring**

In principle, periodic monitoring is conducted twice a year to determine whether the study remains safe, is being conducted in accordance with the study protocol, and whether the data is being recorded correctly.

Centralized monitoring is performed based on data recorded on case report forms (CRFs) accumulated by the data center, and no site monitoring other than checking of original data during site visits is conducted.

Monitoring of the following items is conducted:

- Status of data compilation
- Patient eligibility status
- Protocol treatment/termination status
- Serious adverse events
- Adverse reactions/adverse events
- Protocol deviations
- Overall survivals
- Other problems related to the progress and safety of the examination

Regular monitoring reports prepared by the data center will be submitted to the research representative, the executive committee, and the effect/safety evaluation committee.

If it is determined to be difficult to complete this clinical trial as a result of delays in subject enrollment, frequent protocol deviations, the occurrence of unexpected serious adverse events, clear treatment-related subject death, etc., the study representative will consult with the Data and Safety Monitoring Committee as to whether to continue or terminate the study. The study representative will inform all participating institutions of the decision reached.

# **15. Publication of Study Outcomes**

The results and outcomes obtained from this study shall be shared among all medical institutions participating in the examination and their publication shall be discussed among these parties. All confidential information of subjects shall be protected in the event of publication.

Conference presentations and publications will be prepared and published with the approval of the permanent board of the Japanese Foundation for Multidisciplinary Treatment of Cancer (JFMC) following deliberation of the JFMC Clinical Trial Review Committee.

# **16. Study Organization**

## **16-1. Principal Investigator/Protocol Coordinator**

Naohiro Tomita, Department of Surgery, Hyogo College of Medicine

## **16-2. Executive Committee**

Role: Proposes coordinative activities with relevant hospitals in the area, supports subject enrollment, and oversees study progress.

Hideyuki Ike Dept. of Surgery, Saiseikai Yokohamashi Nanbu Hospital

Hideyuki Ishida Department of Digestive Tract and General Surgery, Hepatology, Saitama Medical Center, Saitama Medical University

Eigo Otsuji Division of Digestive Surgery, Kyoto Prefectural University of Medicine

Norikazu Ohno Department of Surgery, Hyogo Cancer Center

Masazumi Okajima Department of Endoscopic Surgery and Surgical Science, Hiroshima University

Yutaka Ogata Department of Surgery, Kurume University Medical Center

Kiyotaka Okuno Department of Surgery, Kindai University Faculty of Medicine

Masato Kusunoki Department of Gastrointestinal and Pediatric Surgery, Mie University Graduate School of Medicine

Keiji Koda Department of Surgery, Teikyo University Chiba Medical Center

Michiya Kobayashi Department of Human Health and Medical Sciences, Hospital Administration Section, Kochi Medical University Medical School

Takeru Kondo Department of Surgery, NHO Nagoya Medical Center

Yoshiharu Sakai Division of Gastrointestinal Surgery, Faculty of Surgery, Kyoto University Graduate School of Medicine

Kazuaki Sasaki Department of Surgery, Otaru Ekisaikai Hospital

Sotaro Sadahiro Department of Gastrointestinal Surgery, Tokai University

Taro Sato Department of Medical Oncology, Faculty of Medicine, Kinki University

Toshihiko Sato Department of Surgery, Yamagata Prefectural Central Hospital

Kenichi Sugihara Department of Surgical Oncology, Graduate School of Medical and Dental Sciences, Tokyo Medical and Dental University

Yasumasa Takii Department of Surgery, Niigata Cancer Center, Niigata Hospital

Takashi Tsuchiya Depts. of Surgery and Gastrointestinal Surgery, Sendai City Medical Center, Sendai Open Hospital

Naohiro Tomita Faculty of Surgery, Hyogo College of Medicine

Yoshio Naomoto Department of Gastroenterological Surgery, Okayama University Hospital

Motokazu Nishimura Department of Surgery, Kanazawa Red Cross Hospital

Riichiro Nezu Department of Surgery, Osaka Rosai Hospital

Shoichi Hazama Digestive Surgery and Surgical Oncology, Yamaguchi University School of Medicine

Hideo Baba Department of Gastroenterological Surgery, Graduate School of Medical Sciences, Kumamoto University

Takashi Hirai Department of Gastroenterological Surgery, Aichi Cancer Center Central Hospital

Tomohisa Furuhata 1st Dept.of Surgery, Sapporo Medical University School of Medicine

Narikazu Boku Department of Gastroenterological Surgery, Shizuoka Cancer Center

Kotaro Maeda Department of Gastroenterological Surgery, Fujita Health University

Yoshihiko Maebara Department of Surgery and Science, Graduate School of Medical Sciences, Kyushu University

Hideyuki Mishima Department of Surgery, NHO Osaka National Hospital

Hiroyuki Masuko Department of Surgery, Sapporo Kosei Hospital

Takayuki Morita Department of Surgery, Aomori Prefectural Central Hospital

Masaki Mori Department of Gastroenterological Surgery, Graduate School of Medicine, Osaka University

Shigeki Yamaguchi Department of Lower Gastroenteral Surgery, Saitama Medical University International Medical Center

Kazuhiro Yoshida Department of Surgical Oncology, Gifu University Graduate School of Medicine

Masahiko Watanabe Department of Surgery, Kitasato University School of Medicine

## **16-3. Research Management Investigators**

Role: Assists the Principal Investigator and manages the progress of subject enrollment, protocol treatment initiation and follow-up surveys, and responds to inquiries from participating facilities and the study secretariat. Evaluates emergency adverse event reports and facilitates prompt countermeasures as necessary

Sotaro Sadahiro, Department of Gastrointestinal Surgery, Tokai University

Hideyuki Mishima, Department of Surgery, NHO Osaka National Hospital

## **16-4. Data and Safety Monitoring Committee**

Role: Evaluates the results of periodic monitoring reports and interim analyses prepared by the data center and makes necessary recommendations. Evaluates the content of emergency adverse event reports and makes necessary recommendations.

Kei Muro, Department of Clinical Oncology, Aichi Cancer Center Hospital

Noboru Oriuchi, Department of Nuclear Medicine, Graduate School of Medicine, Gunma University

Satoshi Morita, Department of Biostatistics and Epidemiology, Yokohama City University Medical Center

## **16-5. Supervising Statistician**

Role: Drafts statistical content related to study/protocol design. During interim and final analyses, provides expert guidance to the data center responsible for data analysis and reviews the results.

Chikuma Hamada, Department of Industrial Administration, Tokyo University of Science

## **16-6. HRQOL/Medical Economics Review Committee**

Role: Protocol development and results analysis/assessment with respect to the tertiary HRQOL/medical economics study conducted at some study sites.

Takashi Fukuda, Department of Clinical Epidemiology & Health Economics, School of Public Health, The University of Tokyo

Takeru Shiroiwa, Department of Drug Policy and Management, School of Public Health, The University of Tokyo

Megumi Ishiguro, Department of Surgical Oncology, Graduate School of Medical and Dental Sciences, Tokyo Medical and Dental University

## **16-7. Participating Study Sites**

Approximately 300 sites (APPENDIX 1)

## **16-8. Study Secretariat**

Study Secretariat: Japanese Foundation for Multidisciplinary Treatment of Cancer

Tani Bldg., 3F, 1-28-6 Kameido, Koto-ku, Tokyo, Japan 136-0071

TEL: 03-3239-2011 (data center)

03-3239-2341 (study representative/administration)

0120-184100 (free dial)

FAX: 03-3239-2553

E-mail: jfmc-dc@jfmc.or.jp (data center)

jfmc@jfmc.or.jp (study representative/administration)

Please use the following contact information until September 12, 2008 (no changes to TEL, FAX, or E-mail information):

Chiyoda K Bldg., 4F, Kudan-minami 3-7-14, Chiyoda-ku, Tokyo, Japan 102-0074

# **17. References**

1. Cancer Information Service (Home page, Center for Cancer Control and Information Services, National Cancer Center). http://ganjoho.ncc.go.jp/professional/index.html.
2. Muto T, Kotake K, Koyama Y. Colorectal cancer statistics in Japan: data from JSCCR registration, 1974-1993. Int J Clin Oncol. 2001 Aug; 6 (4): 171-6.
3. Haller DG, Catalano PJ, Macdonald JS, O'Rourke MA, Frontiera MS, Jackson DV, et al. Phase III study of fluorouracil, leucovorin, and levamisole in high-risk stage II and III colon cancer: final report of Intergroup 0089. J Clin Oncol. 2005 Dec 1;23 (34):8671-8.
4. Comparison of flourouracil with additional levamisole, higher-dose folinic acid, or both, as adjuvant chemotherapy for colorectal cancer: a randomised trial. QUASAR Collaborative Group. Lancet. 2000 May 6;355 (9215):1588-96.
5. Andre T, Quinaux E, Louvet C, Gamelin E, Bouche O, Achille E, et al.: Updated results at 6 year of the GERCOR C96.1 phase III study comparing LV5FU2 to monthly 5FU-leucovorin (mFufol) as adjuvant treatment for Dukes’ B2 and C colon cancer patients. J Clin Oncol, ASCO Annual Meeting Proceedings. 2005;23(16)(Suppl 1):251s(abstr 3522)
6. Andre T, Colin P, Louvet C, Gamelin E, Bouche O, Achille E, et al. Semimonthly versus monthly regimen of fluorouracil and leucovorin administered for 24 or 36 weeks as adjuvant therapy in stage II and III colon cancer: results of a randomized trial. J Clin Oncol. 2003 Aug 1;21 (15):2896-903.
7. Saini A, Norman AR, Cunningham D, Chau I, Hill M, Tait D, et al. Twelve weeks of protracted venous infusion of fluorouracil (5-FU) is as effective as 6 months of bolus 5-FU and folinic acid as adjuvant treatment in colorectal cancer. Br J Cancer. 2003 Jun 16;88 (12):1859-65.
8. Lembersky BC, Wieand HS, Petrelli NJ, O'Connell MJ, Colangelo LH, Smith RE, et al. Oral uracil and tegafur plus leucovorin compared with intravenous fluorouracil and leucovorin in stage II and III carcinoma of the colon: results from National Surgical Adjuvant Breast and Bowel Project Protocol C-06. J Clin Oncol. 2006 May 1;24 (13):2059-64.
9. Twelves C, Wong A, Nowacki MP et al. Capecitabine as adjuvant treatment for Stage III colon cancer. N. Engl. J. Med. 352 (26), 2696-2704 (2005).
10. Saltz LB, Niedzwiecki D, Hollis D, Goldberg RM, Hantel A, Thomas JP, et al. Irinotecan plus fluorouracil/leucovorin (IFL) versus fluorouracil/leucovorin alone (FL) in stage III colon cancer (intergroup trial CALGB C89803). J Clin Oncol, ASCO Annual Meeting Proceedings. 2004;22(14)(Suppl 15):245s(abstr 3500)
11. Ychou M, Raoul J, Douillard J, Bugat R, Mineur L, Viret F, et al. A phase III randomized trial of LV5FU2+CPT-11 vs. LV5FU2 alone in adjuvant high risk colon cancer (FNCLCC Accord02/FFCD9802). J Clin Oncol, ASCO Annual Meeting Proceedings. 2005;23(16)(Suppl 1):246s(abstr3502)
12. Van Cutsem E, Labianca R, Hossfeld D, Bodoky G, Roth A, Aranda E, et al. Randomized phase III trial comparing infused irinotecan/5-fluorouracil (5-FU)/folinic acid (IF) versus 5-FU/FA (F) in stage III colon cancer patients (pts). (PETACC 3). J Clin Oncol, ASCO Annual Meeting Proceedings. 2005;23(16)(Suppl 1):3s(abstr LBA8)
13. Andre T, Boni C, Mounedji-Boudiaf L, Navarro M, Tabernero J, Hickish T, et al. Oxaliplatin, fluorouracil, and leucovorin as adjuvant treatment for colon cancer. N Engl J Med. 2004 Jun 3;350 (23):2343-51.
14. Wolmark N, Wieand HS, Kuebler JP, Colangelo L, Smith RE. A phase III trial comparing FULV to FULV + oxaliplatin in stage II or III carcinoma of the colon: Results of NSABP Protocol C-07. J Clin Oncol, ASCO Annual Meeting Proceedings. 2005;23(16)(Suppl 1):246s(abstr LBA3500)
15. Sakamoto J, Ohashi Y, Hamada C, Buyse M, Burzykowski T, Piedbois P. Efficacy of oral adjuvant therapy after resection of colorectal cancer: 5-year results from three randomized trials. J Clin Oncol. 2004 Feb 1;22 (3):484-92.
16. Wolmark N, Rockette H, Mamounas E, et al. Clinical trial to assess the relative efficacy of fluorouracil and leucovorin, fluorouracil and levamisole, and fluorouracil, leucovorin, and levamisole in patients with Dukes’ B and C carcinoma of the colon: results from National Surgical Adjuvant Breast and Bowel Project C-04. J Clin Oncol 1999, 17: 3553-3559.
17. Scheithauer W, McKendrick J, Begbie S, Borner M, et al.; X-ACT Study Group. Oral capecitabine as an alternative to i.v. 5-fluorouracil-based adjuvant therapy for colon cancer: safety results of a randomized, phase III trial. Ann Oncol. 2003; 14: 1735-43.
18. Cassidy J, Scheithauer W, McKendrick J, et al. Capecitabine (X) vs bolus 5-FU/leucovorin as adjuvant treatment for colon cancer (the X-Act Study): positive efficacy results of a phase III trial. J Clin Oncol 2004; 22 (suppl): 247s, abstr 3509.
19. Hoff PM, Ansari R, Batist G, et al. Comparison of oral capecitabine versus intravenous Fluorouracil plus Leucovorin as first line treatment in 605 patients with Metastatic colorectal cancer: results of a randomized phase III study. J Clin Oncol 2001; 19: 2282-2292.
20. Van Cutsem E, Twelves C, Cassidy J, et al. Oral capecitabine compared with intravenous Fluorouracil plus Leucovorin in patients with Metastatic colorectal cancer: results of a large phase III study. J Clin Oncol 2001, 19, 4097-4106.
21. Van Cutsem E, Hoff PM, Harper P, et al. Oral capecitabine vs intravenous 5-FU and leucovorin: integrated efficacy data and novel analyses from two large randomized, phase III trials. Br J Cancer 2004; 90: 1190–1197.
22. Hyoudo I, et al. A phase II Study of the global dose and schedule of capecitabine in Japanese patients with metastatic colorectal cancer. Jpn J Clin Oncol.; 2006 Jul; 36(7):410-7.
23. Nagore E. Antineoplastic Therapy-Induced Palmar Plantar Erythrodysesthesia ('Hand-Foot') Syndrome: Incidence, Recognition and Management. Am J Clin Dermatol.; 2000 Jul-Aug: 1 (4): 225-234.
24. S. Lee et al., Pyridoxine is not effective for the prevention of hand foot syndrome (HFS) associated with capecitabine therapy: Results of a randomized double-blind placebo-controlled study. Journal of Clinical Oncology; 2007 ASCO Annual Meeting Proceedings Part I. Vol 25, No. 18S (June 20 Supplement), 2007: 9007
25. Katayose Y, et al.: The Journal of Japan Society for Cancer Therapy; 40(2): 707, 2005.
26. Kondo N, et al.: Program Abstracts. 14th Convention of the Japanese Breast Cancer Society: 335, 2006.
27. Nakano Y, et al. Journal of the Japan Surgical Society. Vol.108, Special Issue No. 2 (Sup. 2): 657, 2007.
28. Takashima T, et al.: Program Abstracts. 15th Convention of the Japanese Breast Cancer Society: 294, 2007.
29. Kato T, et al.: Program Abstracts. 15th Convention of the Japanese Breast Cancer Society: 377, 2007.
30. Fujii C, et al.: Program Abstracts. 15th Convention of the Japanese Breast Cancer Society: 439, 2007.
31. R. Simon et al., Randomized PhaseII Clinical Trials, Cancer Treat Rep, 1985, 69:1375-81
32. Lan K, Demets L: Discrete sequential boundaries for clinical trials. Biometrika 70:659-663, 1996.
